# Effect of ultrasonic surface impact on the microstructural characterization and mechanical properties of 316L austenitic stainless steel

**DOI:** 10.1371/journal.pone.0307400

**Published:** 2024-07-25

**Authors:** Jiangpei Zhu, Mei-Ling Zhuang, Yuting Qi, Bin Chen, Xiaojian Cao

**Affiliations:** 1 School of Transportation and Civil Engineering, Nantong University, Nantong, China; 2 School of Civil Engineering, Nantong Institute of Technology, Nantong, China; 3 School of Civil Engineering, Shandong University, Jinan, China; Semnan University, ISLAMIC REPUBLIC OF IRAN

## Abstract

In the present study, effect of ultrasonic impact treatment (UIT) on the microstructural characterization and mechanical properties of 316L stainless steel (hereinafter referred to as 316L) was investigated experimentally. The fatigue fracture mechanism of 316L before and after UIT was revealed. The experimental results indicated that the martensitic grain size induced at the impact edge was about 2.00 Å. The surface modified 316L formed a gradient nanostructure and induced a martensitic phase transformation. The hardness of the surface layer of the modified 316L was twice the hardness of its matrix. The tensile strengths of 316L before and after UIT were 576 MPa and 703 MPa, respectively. The stretching stripes of 316L were more disordered after UIT. The fatigue strengths of 316L before and after UIT were 267 MPa and 327 MPa, respectively. The fatigue cracking of 316L started from the austenite grain boundaries. The fatigue fracture surface was relatively rough. The fatigue crack sources of the modified 316L came from internal inclusions. The inclusions were oxides dominated by SiO_2_. As the stress range increased, the crack initiation site migrated to the interior and the fatigue fracture surface became flatter.

## 1 Introduction

316L austenitic stainless steel has been widely used in engineering because of its high strength, good toughness and corrosion resistance [1]. However, engineering accidents caused by fatigue fracture of metal components have been frequently reported in recent years. Since academician Lu Ke from Institute of Metals, Chinese Academy of Sciences proposed surface nanosizing [2], surface nanosizing has become one of the hot spots in materials science research in the last two decades [2]. Ultrasonic surface impact technology can effectively improve the metal wear resistance [3], fatigue strength [4], corrosion resistance [5] and other properties of materials. Combining the advantages of stainless steel and the frontier of the discipline, it is of great theoretical and engineering significance to explore the mechanical properties and fatigue fracture mechanism of modified 316L austenitic stainless steel.

The surface of the specimen after ultrasonic impact treatment (UIT) will form a gradient deformation layer of certain depth, including surface amorphous and ultrafine crystals [6]. Gradient nanostructured metallic materials have surface grain sizes in the nanometer range, with internal grain sizes gradually increasing to the micrometer range. They have been extensively studied in recent years due to their superior properties over uniform coarse or nanostructured metallic materials [7–19]. Martensitic stainless steels [20] and bearing steels [21] with gradient nanostructures both have high fatigue strength and fatigue life under different loading modes. The fatigue strength of 316L austenitic stainless steel with gradient nanostructures was improved in both high-cycle and low-cycle fatigue states compared to coarse crystalline [22, 23]. Dang et al. [24] found that the tensile strength of the treated 300M steel increased by 3.8%, the low cycle fatigue life was extended by 0.5 times, and the high cycle fatigue life was extended by more than 21 times. Liu et al. [25] investigated the effect of different fatigue cycle intervals on the S690QL of different fatigue cycle intervals in a room temperature. Mordyuk et al. [26] used a combination of UIT and EDM surface alloying of mild steel 20GL and found that the fatigue strength was increased by 15~ 30%. Andrade et al. [27] utilized L-DED additive manufacturing of 316L stainless steel, which showed a 35% increase in fatigue strength. Yan et al. [28] found that ultrasonically rolled can increase the endurance limit of the material by more than 50%. Zhou et al. [29] found that the fatigue life of the treated specimens with conventional shot peening was higher at low stress amplitude (±0.5%) and lower at high stress amplitude (±1.25%). Pramod et al. [30] investigated the low peripheral fatigue of Ti-13Nb-13Zr under ultrasonic shot peening and de-stressing conditions, and found that the ultrasonic rolling can increase the material endurance limit by more than 50%. Pramod Kumar et al. [30] investigated the low cycle fatigue behavior of Ti-13Nb-13Zr under ultrasonic shot peening and de-stressing conditions and found that the fatigue life of the material increased. Hitoshi [31] found that the fatigue strength was the highest when the fatigue strength was peened by cavitation. Zheng et al. [32] found that the fatigue life of the treated specimens using ultrasonic impact and cavitation jet peening can be increased by up to 163.3%. Chen et al. [33] investigated the tensile properties of nanocrystalline 316L austenitic stainless steel experimentally. The results showed that the nanocrystalline samples had a very high yield strength of 1450 MPa. In previous study, Cao et al. [4] found that the nanocrystalline surface layer had the effect of retarding fatigue crack sprouting. After UIT, Kudryavtsev et al. [34] observed that the grains in this depth range were refined by repeated impacts, the strength and surface hardness of the material were improved. Meng et al. [35] observed obvious plastic deforming layer and nanocrystalline grains after UIT. Wang et al. [36] investigated the surface properties of CrCoNi medium-entropy alloy coatings after UIT. The experimental results indicated that the metal surface grains will be refined after UIT, resulting in more twins and dislocations. The twins can effectively inhibit the generation of slip bands and cracks, which can comprehensively improve the hardness, strength and fatigue resistance of metal materials. In summary, UIT can make the hardness, roughness, corrosion resistance, yield strength, tensile strength of metal materials have a significant increase, will make the surface of the metal material grain refinement, and promote the austenite to martensite phase transition. After treatment of the specimen, fatigue strength and fatigue life will also be effectively improved.

UIT involves many parameters. Stress ratio, average stress, stress amplitude, frequency, etc. are factors that affect fatigue life. However, previous studies rarely have hardness, fatigue, scanning electron microscopy, transmission electron microscopy and other tests to systematically analyze, and the analysis of the fatigue section only focuses on the cross-section. To address the above issues, UIT technology was used to surface modify 316L austenitic stainless steel. The effect of UIT on the mechanical properties of 316L austenitic stainless steel was investigated by combining XRD test, tensile and fatigue tests, SEM and transmission electron microscopy investigations,. The fatigue fracture mechanism of 316L austenitic stainless steel before and after UIT was also revealed.

## 2 Materials and experimental procedure

### 2.1 Materials and specimens

**Fig 1:**
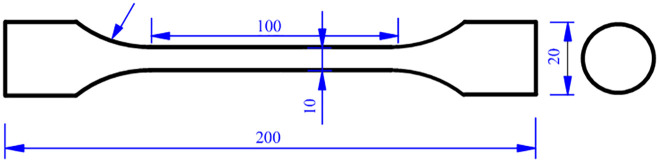
Dimensions of the tensile and fatigue specimens (Unit: mm).

**Fig 2:**
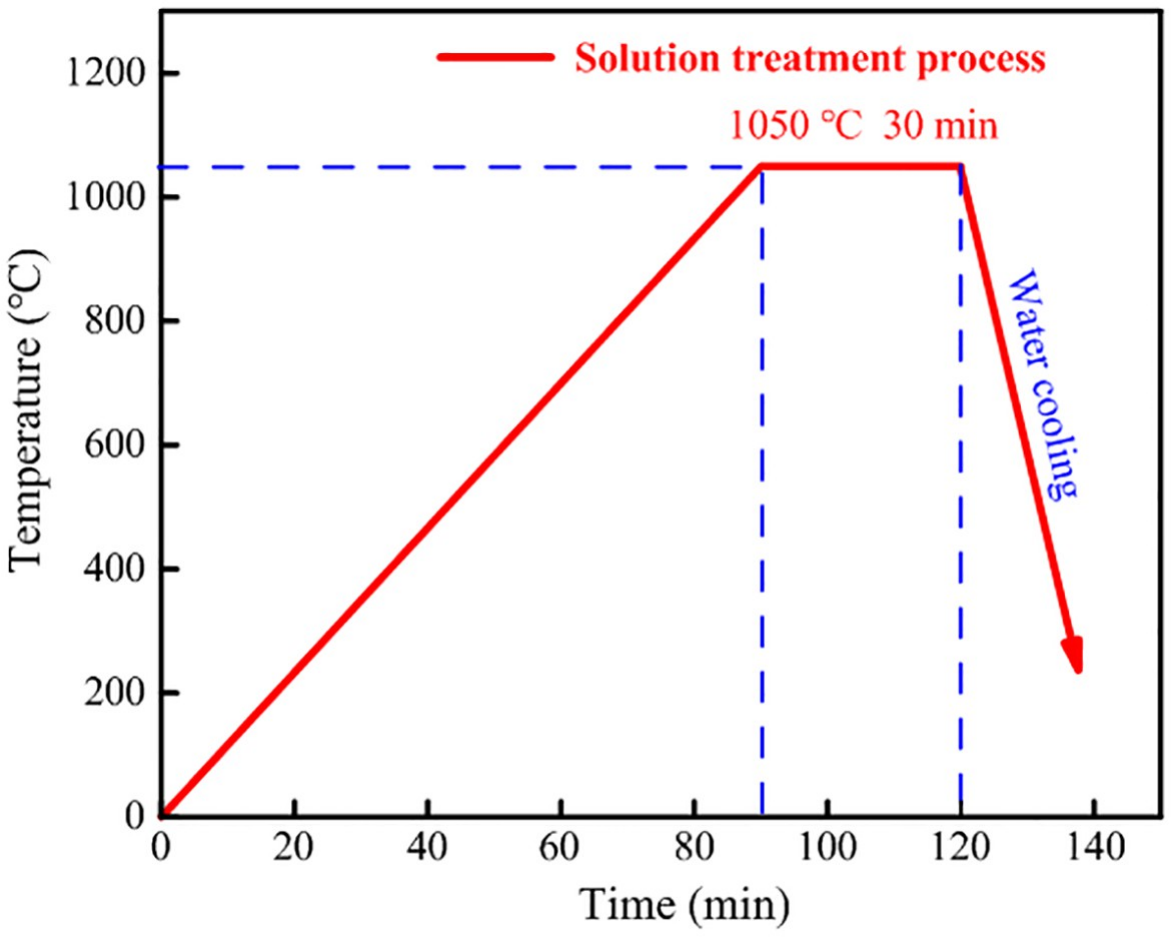
Solid solution treatment parameters of 316L.

The specimens were designed according to ASTM E466 [15] (see Fig 1). The specimen material was Guangyuan 316L austenitic stainless steel (AISI 316L), produced by Taizhou Hualu Stainless Steel Products Factory, China. According to the chemical composition test report of the 316L austenitic stainless steel, its chemical composition is obtained in Table 1. In order to obtain a stable and homogeneous single-phase austenite organization, it was necessary to eliminate the stresses during processing of the material and to dissolve the carbides that may be present, so the material was solid solution treated using all-in-one intelligent muffle furnace. The treated parameters are shown in Fig 2. The material was heat treated at 1050°C for 0.5 h, followed by water quenching. The diameters of the specimens in the loading zone and the test zone were 20 mm and 10 mm, respectively.

**Table 1:** Chemical composition content of 316L austenitic stainless steel.

Chemical composition	C	Si	Mn	P	S	Ni	Cr	Mo	Fe
Content (%)	0.022	0.38	1.03	0.039	0.001	10.05	16.30	2.01	70.168

### 2.2 Ultrasonic nanocrystal surface modification

**Fig 3:**
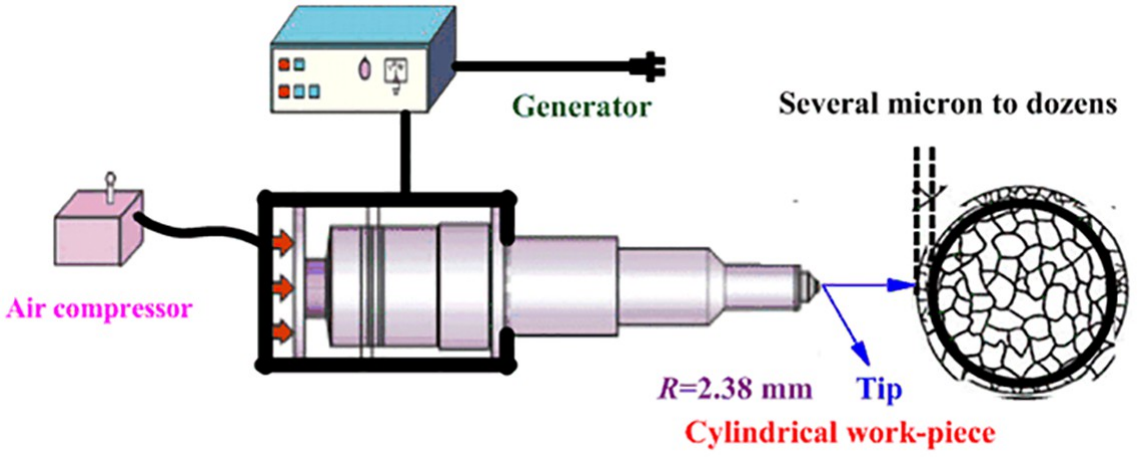
Equipment of ultrasonic impact treatment from Fig 1 in the reference [4].

The UIT equipment is shown in Fig 3, which is from Fig 1 in the reference [4]. The UIT technology generated longitudinal vibrations by means of a piezoelectric ceramic transducer, which applied tens of thousands of sinusoidal impact dynamic loads per unit square millimeter to the metal surface. In addition, a static load was applied by means of an air compressor, so that the total load applied to the surface of the specimen was the sum of the dynamic and static loads. The spherical impact head was made of tungsten carbide (hardness close to diamond). and the heat generated during impact was cooled by high-pressure air and lubricated by kerosene. The number of impacts of the ball head was 50, 000 cycles/mm^2^. The UIT caused severe plastic deformation (several microns to tens of microns) of the stainless steel surface layer and induced nanocrystalline structural surfaces [4]. According to the reference [16], to achieve desirable results, the following parameters can be applied. The number of ultrasonic impact cycles per unit surface area of the specimen is 10,000, the relevant frequency is 30, 000, pin diameter is 2.38 mm. The amount of static load from the air compressor is 0 N. In this paper, modified 316L denotes 316L austenitic stainless steel after UIT; 316L denotes 316L austenitic stainless steel.

### 2.3 Metallographic and hardness tests

Metallographic observations were made on 316L and modified 316L samples. The metallographic specimens were first mounted with a Struers CitoPress mounting machine. The metallographic samples were then polished with a Struers Tegramin-25 grinding and polishing machine. 30 ml of hydrochloric acid, 10 ml of nitric acid 10 ml and 10 ml of water were prepared to form aqua regia. Then, tissue etching was carried out with aqua regia solution and a few drops of ferric chloride solution was added to the aqua regia solution. Because the austenitic sample contains Cr element, Cr would be in the form of oxide film attached to the surface of stainless steel to play the role of corrosion resistance. The role of chloride ions is to destroy the oxide film formed by the Cr element, thus making the austenite visible. The corrosion time was 10 s. Finally, the corrosion was observed and photographed with Zeiss Axio Imager 2 microscope, if the metallographic organization was not obvious, the above steps were repeated and if the metallographic organization was better, it was photographed again under a Gemini SEM 300 scanning electron microscope.

The hardness of the samples was tested on a TMVS-1 digital micro Vickers hardness tester with a force load *F* = 1.962 N and a holding time of 15 s. The hardness symbol for this condition was noted as HV_0.2_. Hardness test were performed on 316L and modified 316L. Three points were made in the middle of the sample. The size of the indentation was measured, and the average of the three points was taken as the matrix hardness of the material using Eq (1) [37].

$${\text{HV}}_{0.2}=0.102e\frac{F}{S}=0.102e\frac{2F\;\text{sin}\;\frac{\alpha }{2}}{d^{2}}$$ (1)

where *F* is the load (Newton force), *S* is the surface area of the indentation (square mm), *α* is the angle of the indenter relative to the surface and *α* = 136°, and *d* is the average diagonal length of the indentation (mm).

### 2.4 XRD test

The modified 316L surface EPS was carried out using an X-ray diffractometer model EMPYREAN from Panacor, Netherlands to characterize the surface organization and structure of modified 316L. The surface of modified 316L was characterized using a Thermo Scientific transmission electron microscope to obtain the atomic ordering, diffraction rings and lattice constants of the impact layer of modified 316L.

### 2.5 Tensile and fatigue test

Tensile tests were performed on the specimens (see Fig 1) using a MTS-200kN universal testing machine at a rate of 1 mm/minute. The tensile properties such as tensile strength, yield strength, shrinkage and elongation of 316L austenitic stainless steel before and after UIT were tested. The numbers of 316L austenitic stainless steel specimens before and after UIT are three. The average values of the three specimens are taken as the test results. The test methods were in accordance with GB/T228.1–2010 2010 [38].

Fatigue tests were performed on the specimens (see Fig 1) using a GPS-100 electromagnetic resonance high-frequency fatigue teste. The tests were performed with sinusoidal loading and the stress ratio *r* was taken as 0.2. The load frequency was 100 Hz. The loaded stress levels are listed in Tables 2 and 3.

**Table 2:** Loading stress levels of 316L.

No.	σ_max_ (MPa)	σ_min_ (MPa)	*S/*2 (MPa)
1	333.33	66.67	133.33
2	341.67	68.33	136.67
3	350.00	70.00	140.00
4	358.33	71.67	143.33
5	366.67	73.33	146.67
6	366.67	73.33	146.67
7	375.00	75.00	150.00
8	375.00	75.00	150.00
9	383.33	76.67	153.33
10	391.67	78.33	156.67
11	466.67	93.33	186.67

Note: *S* is the stress range of the test specimen, and *S* = *σ*_max_ − *σ*_min_.

**Table 3:** Loading stress levels of modified 316L.

No.	*σ*_max_ (MPa)	*σ*_min_ (MPa)	*S* /2 (MPa)
1	408.33	81.67	163.33
2	416.67	83.33	166.67
3	425.00	85.00	170.00
4	433.33	86.67	173.33
5	441.67	88.33	176.67
6	450.00	90.00	180.00
7	458.33	91.67	183.33
8	466.67	93.33	186.67
9	475.00	95.00	190.00
10	483.33	96.67	193.33
11	491.67	98.33	196.67

### 2.6 SEM investigation

The specimens after tensile and fatigue fracture were cut and sampled for fracture observation using a Gemini SEM 300, and scanned for EPS using the SEM’s own OXFORD-50 energy spectrometer, to analyze the cause of the fracture, the fracture mode, the expansion rate, and the expansion characteristics of the material.

## 3 Results and discussion

### 3.1 Metallographic structure and ultrasonic impact surface characterization

**Fig 4:**
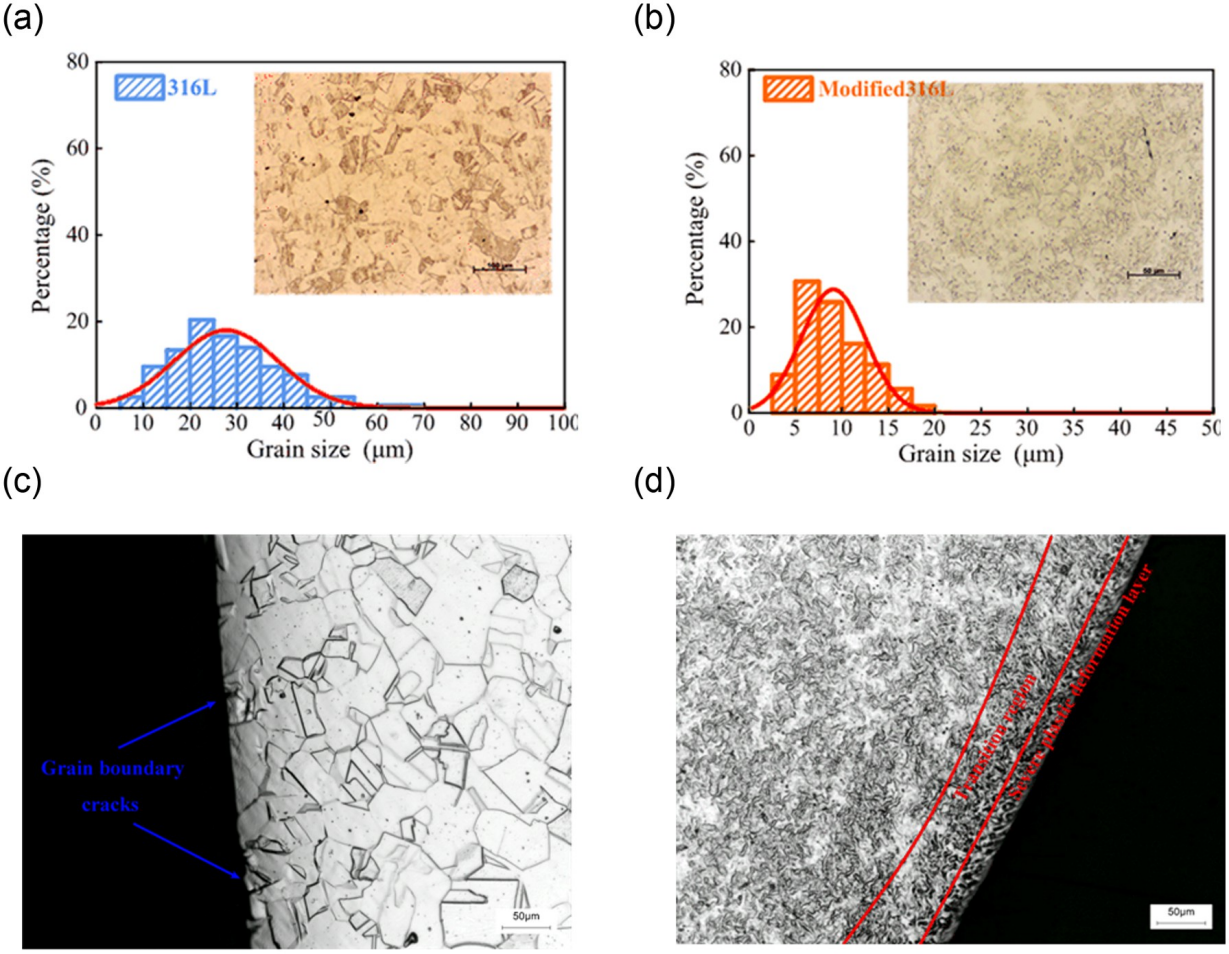
Metallographic structures of 316L and modified 316L specimens at different positions: (a) Matrix of the 316L specimen; (b) Matrix of the modified 316L specimen; (c) Edge of the 316L specimen; (d) Edge of the modified 316Lspecimen.

The middle and edge of the metallographic specimen cross-section were observed using an optical microscope (OM), as shown in Fig 4. A stable homogeneous single phase austenite organization with austenite twin generation in 316L stainless steel can be observed in Fig 4(a). The specimens also contained only a single austenite phase and twinned phases, but the grain size of the austenite was significantly reduced (see Fig 4(b)). The average values of the grain sizes of 316L and modified 316L were 27.5 μm and 9 μm, respectively. The grain size of 316L was three times larger than that of modified 316L due to grain refinement caused by the UIT. There were austenitic grain boundary cracks at the metallographic edges and the surface of modified 316L was not flat (see Fig 4(c)). A strong plastic deformation layer exists at the edge of modified 316L, no obvious grain boundary existed, and the side of modified 316L was relatively smooth (see Fig 4(d)). The depth of the plastic deformation layer was approximately equal to the results from reference [10]. Compared with the results from reference [11], the thickness of the hardening layer of the treated 316L was deeper and the grain refinement was more obvious.

**Fig 5:**
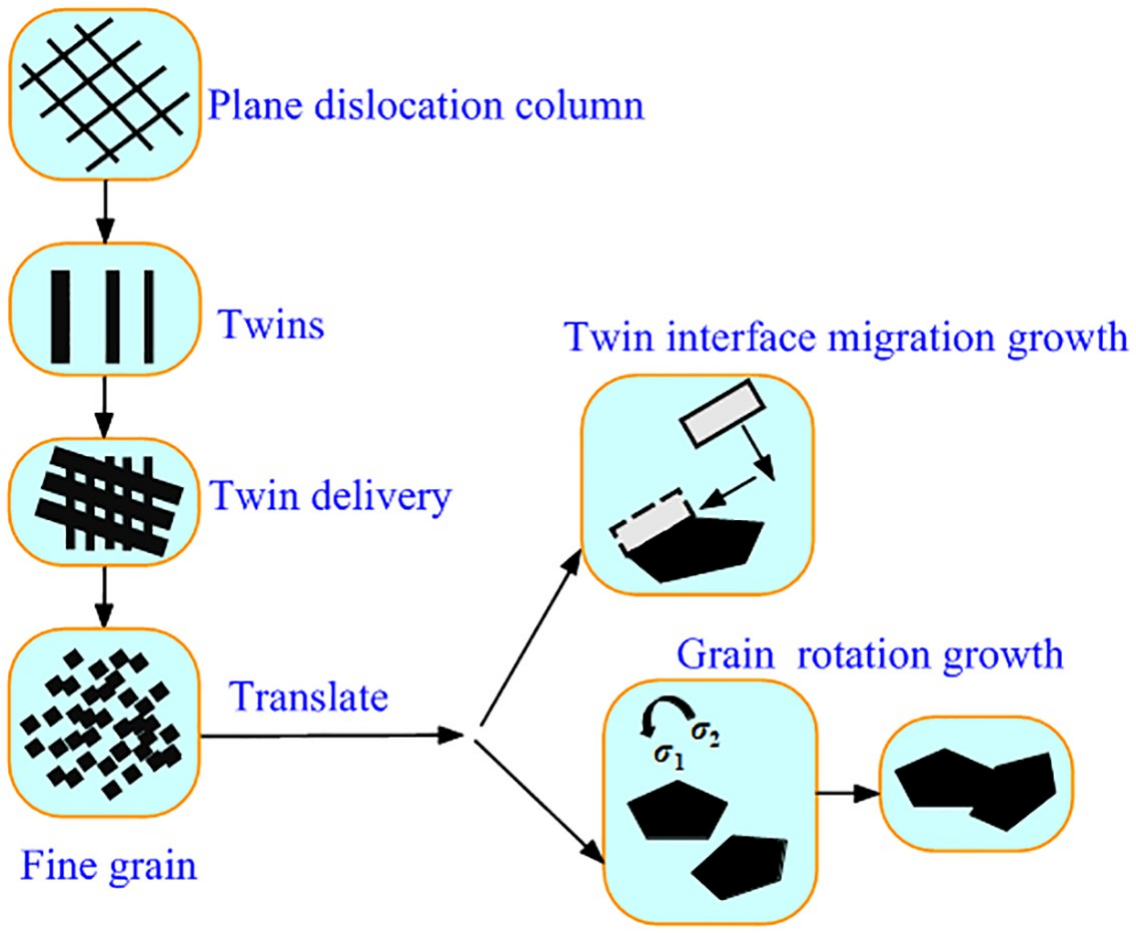
Schematic diagram of the evolution of plastic deformation of modified 316L specimens.

Fig 5 depicts the evolution of plastic deformation of modified 316L. In the ultrasonic impact process of 316L, the diffusion of ultrasonic impact energy to the interior of the crystals was not uniform, the formation of twin crystals was sequential and in different directions, and twin crystals with different orientations would undergo twin crossover during the ultrasonic impact process. The possible causes of grain growth were grain boundary slip or grain boundary torsional deformation.

**Fig 6:**
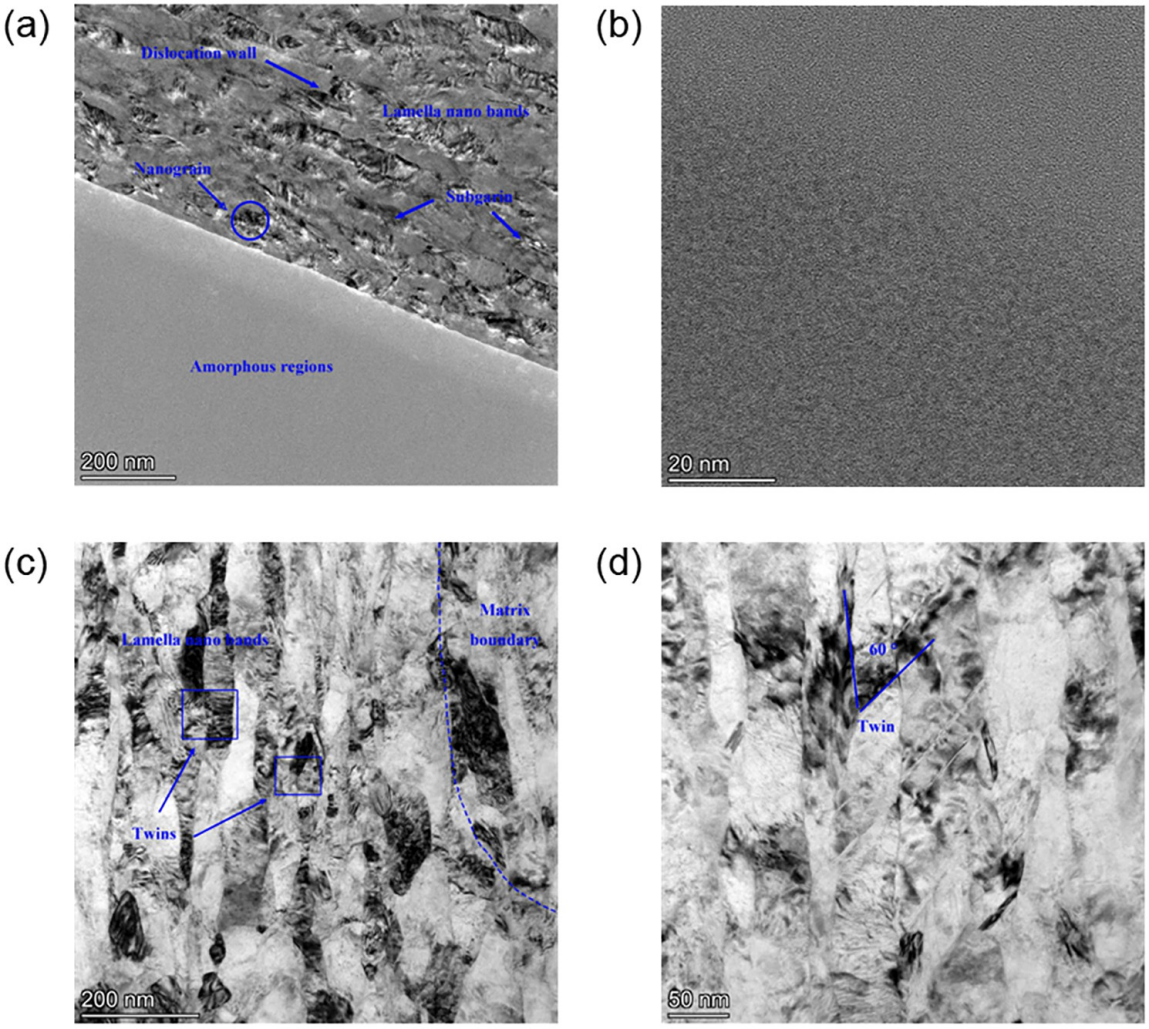
Surface texture and fine crystal organization of modified 316L specimens: (a) Surface texture; (b) Surface amorphous region; (c) Nanocrystalline region; (d) Twin delivery.

To characterize the fine crystal organization of the surface layer of modified 316L, the surface texture of modified 316L was analyzed by transmission electron microscopy as shown in Fig 6. The bright and dark phases of modified 316L can be clearly seen in Fig 6(a). Modified 316L had a multi-phase organization, and its surface grains of modified 316L had been broken into nanocrystals. Nanocrystalline and amorphous regions had been formed on the surface of modified 316L, which had different grain orientations, and some of the grains with favorable orientations firstly started to slip deform, while the grains with unfavorable orientations might still be in the elastic deformation state. Therefore, the amount of plastic deformation under external force was different. These nanocrystalline and amorphous regions exhibited a nested structure, which significantly increased the local surface hardness of modified 316L. It was noteworthy that the surface layer of the crystalline zone in the modified 316L specimen contained a large number of nanoscale particles. As the depth increased, many lamellar sub-grains with uneven morphology and fuzzy boundaries with lamellar nanobelts appeared. Some tiny dislocation walls were also observed between the lamellae. Fig 6(b) shows the amorphous region, which was diffracted on the nanoscale of the beam spot. The diffraction spots appeared as meat splotches, observed using a high-resolution transmission electron microscopy, with the diffracted spot appearing as a fleshy mass (as shown in the upper right corner). The broadened Debye ring was characteristic of the amorphous scattering [39]. Therefore, amorphous bodies were present. The average grain size of the ultrasonically impacted surface layer was about 34.8 nm as can be seen in Fig 6(c). The average size of the refined nanoparticles was in the same order of magnitude as the results from reference [17]. With the increase of the depth, the shear strain and shear strain rate of the specimen by the ultrasonic impact device tended to decrease, and the dislocation density in the deeper layer was lower than that in the surface layer, so it was difficult to form new fine grains in the deeper region. The substrate boundary can be observed in Fig 6(c). As can be seen from Fig 6(d), the internal twinning was full of dislocations, and the orientation difference was the angle formed by the intersection of the two twin systems, which was 60°. Twin 1 and twin 2 were intersecting and interspersed states, but twin 2 did not cross the spacing of twin 1. In summary, the twins arose at grain boundary and extended into the interior of the grains, but never penetrated the entire grain. The dislocation density of the specimens increased after UIT, which was agreement with the findings in reference [16].

**Fig 7:**
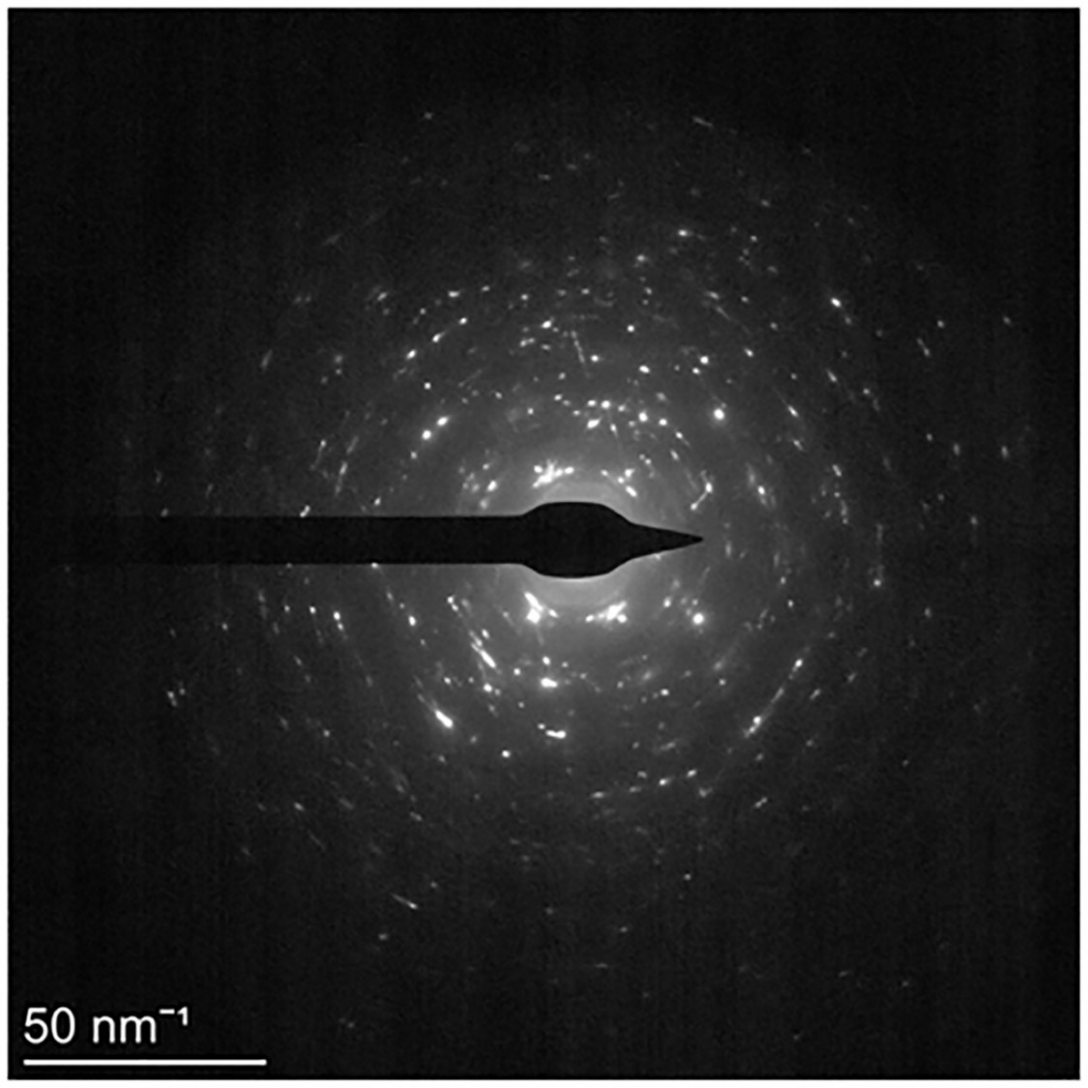
SAED pattern of crystal region of modified 316L specimens.

Fig 7 shows the selected area electron diffraction (SAED) pattern of a crystal region of modified 316L. If the sample contained multiple grains and the grains were oriented completely randomly, then each grain face and each grain orientation had the same chance of diffraction, resulting in the concentric rings shown in Fig 7. The ratio of the radius of the concentric ring was $R_{1}^{2}:R_{2}^{2}:R_{3}^{2}:R_{3}^{2}\cdots =3:4:8:11\cdots$. According to the radius relationship between diffraction rings can be obtained that modified 316L was a face-centered cubic (FCC) structure. Transmission electron micrographs of diffraction analysis of modified 316L using the ring GUI module in CrysTBox software showed that the diffraction indices were *γ*(1 1 1), *γ*(2 0 0), *γ*(3 1 1), *α*(2 1 1), *γ*(2 2 2) ⋯. It was noteworthy that the plastic deformation brought about by the ultrasonic impact induced a martensitic phase transformation of the material, i.e. *α*-Fe induced transformation into *γ*-Fe, which was agreement with the findings in references [13, 15].

### 3.2 Microhardness

**Fig 8:**
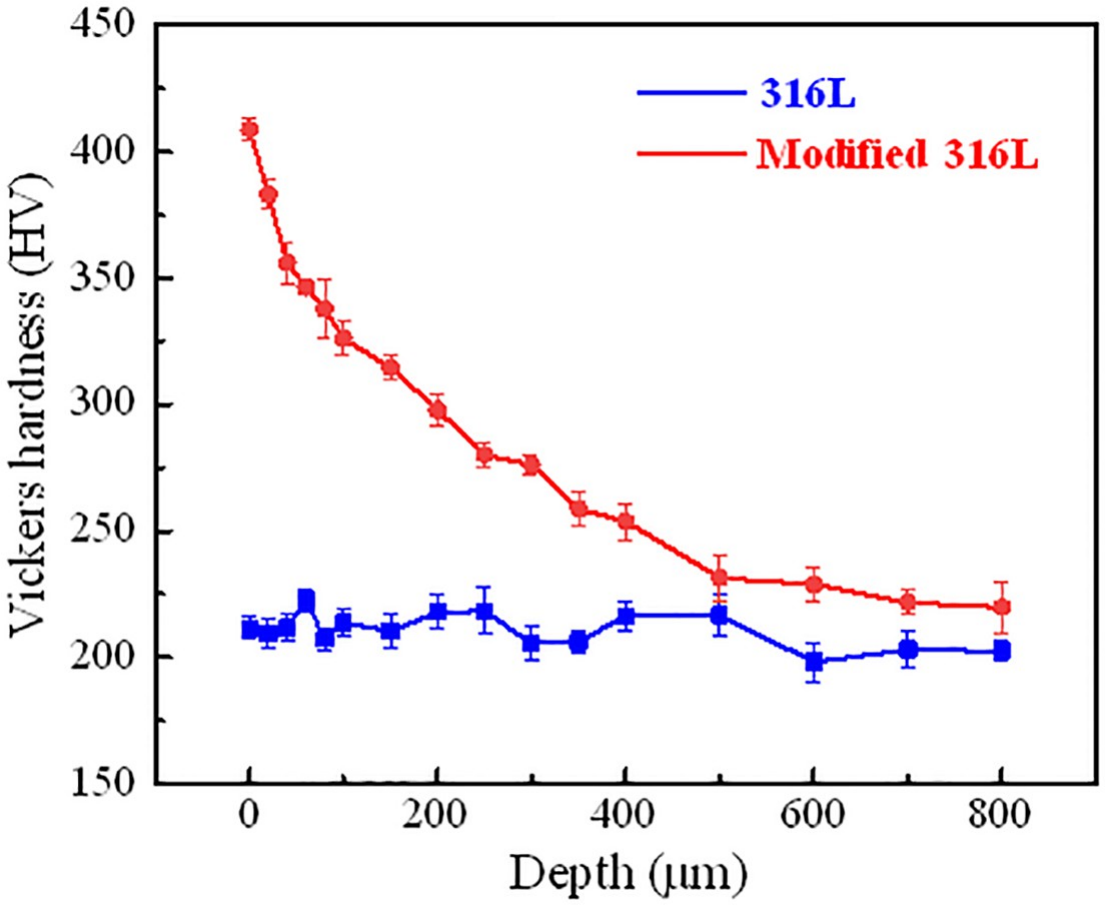
The cross-sectional hardness variation curves of with the depth.

The measured matrix hardnesses of 316L and modified 316L were 208.87 HV and 220.16 HV, respectively. The variation curves of the cross-sectional hardness with the depth (distance from the cross-section to the surface) are described as shown in Fig 8. The hardness of 316L was stable between 200 HV and 220 HV. The hardness of modified 316L surface was up to 428.7 HV, which was twice the hardness of the modified 316L matrix. This was in agreement with the findings in references [14, 16]. When the depth was 0~40 μm, the hardness of modified 316L decreased dramatically. When the depth was 40~400 μm, the decreasing trend of the hardness of modified 316L was slowed down. When the depth was greater than 500 μm, the hardness did not change significantly and stabilized at about 226 HV, which was roughly equivalent to the hardness of 316L matrix. Overall, the hardness of modified 316L was higher than that of 316L. Ultrasonic impact on the metal surface can effectively improve the hardness of 316L. This effect was most pronounced in the range of a few tens of micrometers, up to about 600 μm, which was agreement with the results concluded by Herbster et al. [12]. In accordance with the law reflected in the Hall-patch equation, this explained why the cross-sectional hardness of modified 316L decreased abruptly after a few tens of microns.

### 3.3 XRD diffraction analysis

**Fig 9:**
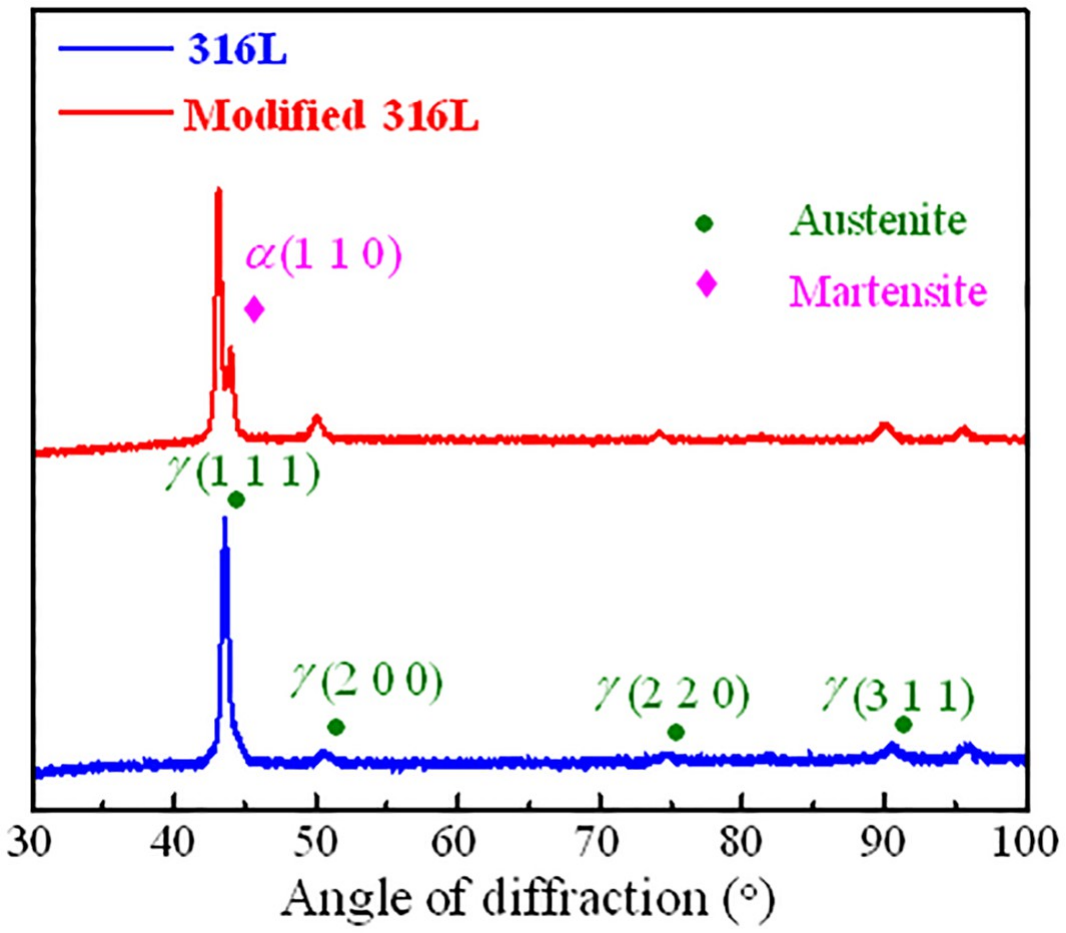
XRD peak spectra of 316 L and modified 316L specimens.

XRD diffraction analysis was performed on 316L and modified 316L, as shown in Fig 9. It can be seen that the martensitic grain size induced at the impact edge was about 2.00 Å. There were four diffraction crystal surfaces for both 316L and modified 316L, which were *γ* (1 1 1), *γ* (2 0 0), *γ* (2 2 0), *γ* (3 1 1). The diffraction index of the transmission electron microscope was consistent with the crystallographic index of XRD, ensuring the accuracy of the XRD diffraction analysis result. Modified 316L sample showed a bifurcation peak on the strongest diffraction peak *α* (1 1 0) and the presence of a very weak characteristic peak *α* (2 1 1), which were found to be martensitic when comparing PDF cards [40, 41], indicating that the surface of the 316L (UI) sample induced martensitic phase transformation during high frequency ultrasonic impact, i.e., *α*-Fe induced transformation to *γ*-Fe [42]. Overall, the characteristic peak of modified 316L sample was shifted to the left.

### 3.4 Tensile properties

**Fig 10:**
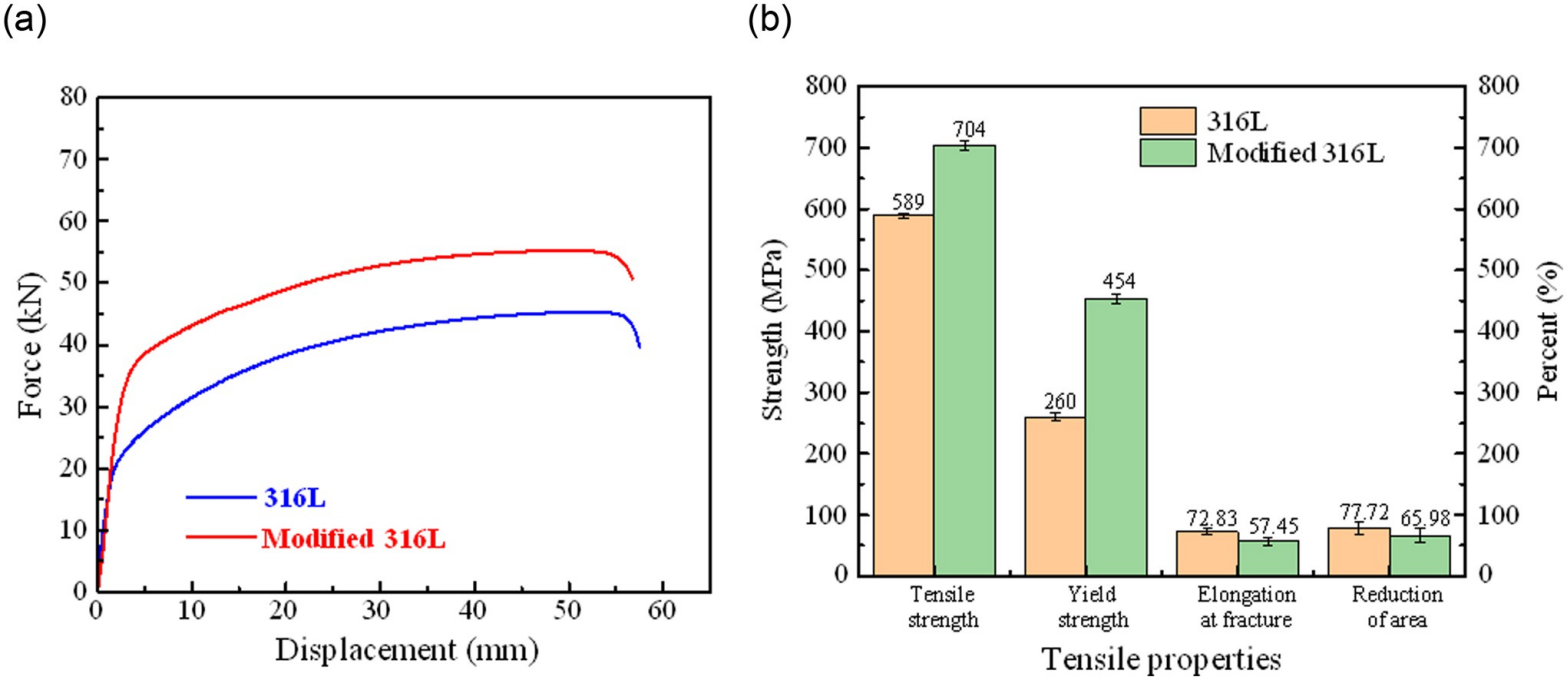
Tensile test results of 316 L and modified 316L specimens: (a) Force displacement curves; (b) Tensile properties.

**Fig 11:**
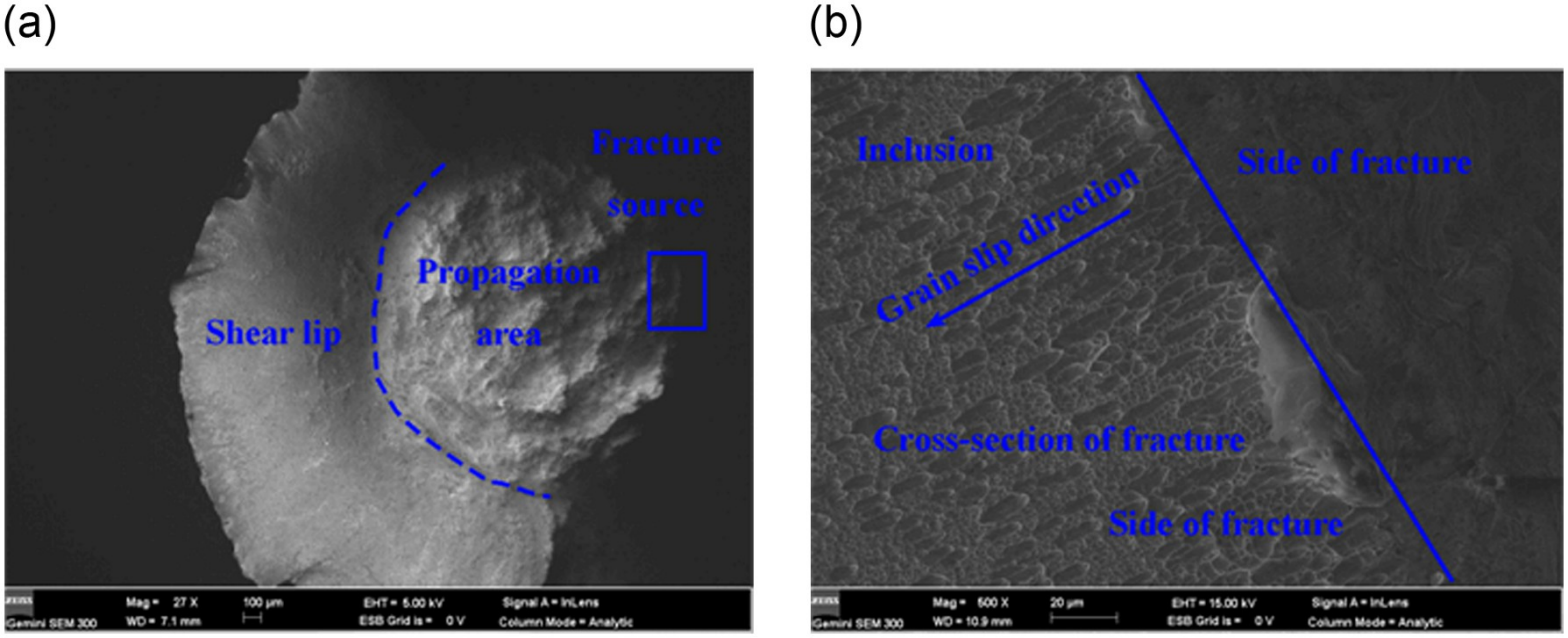
Morphology of tensile fracture morphology of 316L specimens: (a) Full view; (b) Crack initiation site.

**Fig 12:**
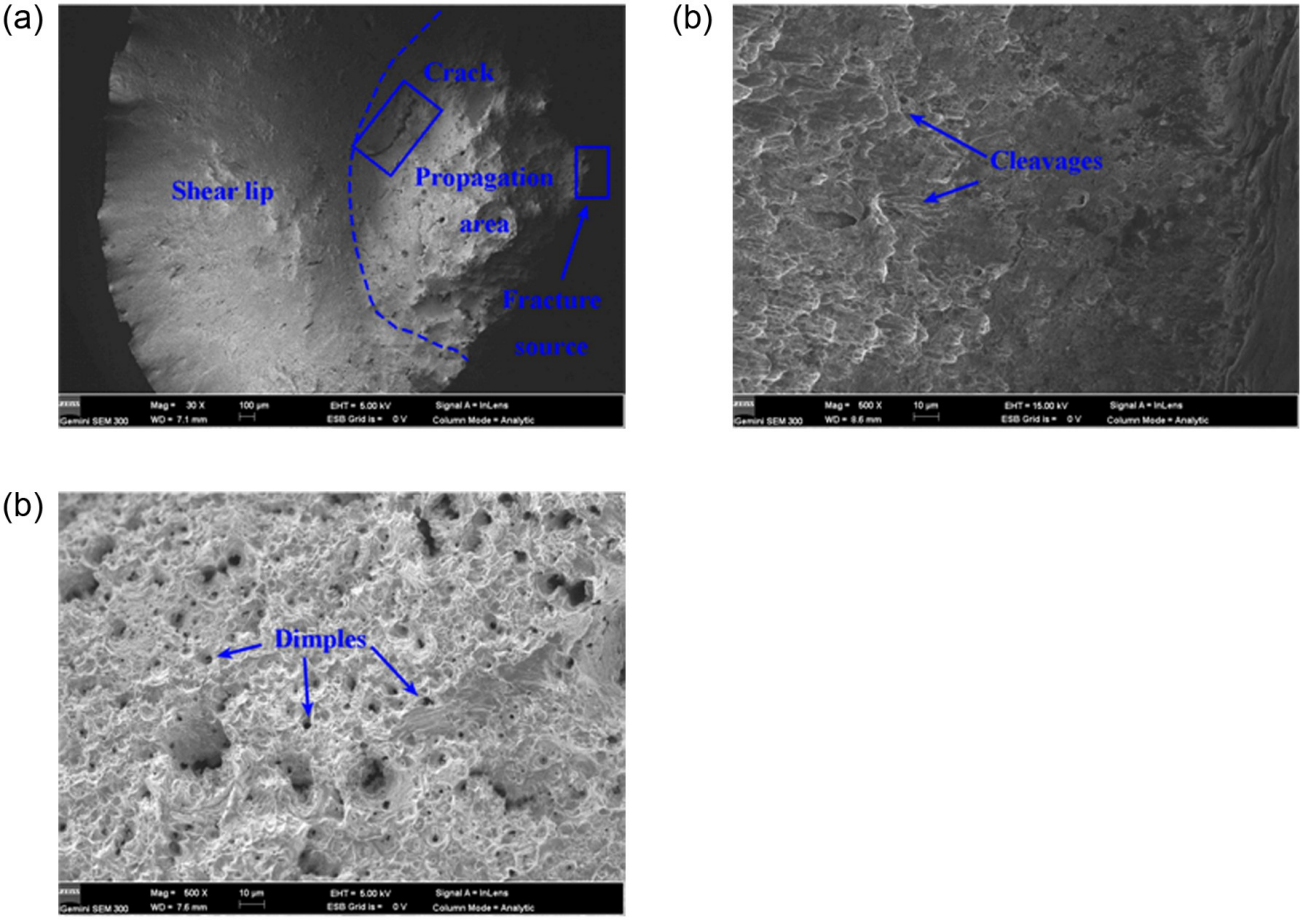
Morphology of tensile fracture morphology of modified 316L specimens: (a) Full view; (b) Crack initiation site; (c) Middle region of fracture surface.

The force-displacement curves of the three 316L (modified 316L) specimens are in better agreement, so the force-displacement curve of only one specimen is selected, as described in Fig 10(a). The tensile properties of 316L and modified 316L specimens are described in Fig 10(b) and Table 4. In Table 4, the tensile results are the average values of the three specimens. After the UIT, the tensile strength of the specimen increased by 19.52%, yield strength increased by 74.61%, and elongation at fracture and reduction of area were decreased by 21.11% and 15.11%, respectively. Compared with the results from reference [16], the increase in yield strength and tensile strength of the modified 316L was slightly increased, but elongation at fracture and reduction of area were decreased. The fracture morphology of 316L specimens under uniaxial tensile loading is shown in Fig 11. The fracture morphology showed a bowl shape (see Fig 11(a)). The fracture pattern was typical of ductile fracture, including three parts: fracture source, extension zone and shear lip. This was a shear fracture that slides opened about 45° in an axial direction at, and was accompanied by a more pronounced necking. As can be seen from Fig 11(b), the stretching stripe distribution of 316L specimen was uniform, and the grain slip distance was stable between 12 and 24 μm, which was a shear dimple for reverse elongation. The crack initiation site of 316L specimen was located at the surface, which has the other material inclusions or grain boundaries. As can be seen from Fig 11(c), 316L specimen also had a more uniform distribution of dimples, which were isometric dimples formed by stretching. The good plasticity of the 316L specimens was demonstrated and confirmed by the high elongation at break of 72.83%. Fig 12 shows the fracture morphology of modified 316L specimens under uniaxial tensile loading. The fracture morphology showed an irregular bowl shape (see Fig 12(b). There was no obvious central rounded bottom, but there was also a shear fracture that slides opened about 45°in an axial direction. Obvious macroscopic cracks appeared at the bottom around the bowl of the specimen. As can be seen from Fig 12(b), the stretching stripe distribution of modified 316L specimen was more disordered, the slip line was torn, and the slip distance was basically stable within 10 μm, which can explained that the elongation and area reduction of modified 316L specimens were small. The material edge of the specimen had no obvious stretching stripes and no other material inclusions. Typical deconvoluted fracture can be observed in the modified 316L specimen near the crack source, indicating that the fracture mode in this region was dominated by brittle fracture. Combined with the full view of modified 316L specimen, it can be determined that the tensile fracture of modified 316L specimen originated from the subsurface layer, i.e., between the nanolayer and the matrix layer. After the matrix basically lost its strength, the cracks propagated outward until modified 316L specimen completely fractured. The reason for this wa the result of the reduction of the elastic-plastic limit of the surface-modified layer. During the UIT, a reinforced layer with high hardness and fine microstructure was produced on the surface of the specimen, which resulted in higher density and lower surface plasticity of the material and the need to counterbalance the compressive stresses in the surface layer with higher tensile stresses. As a result, the material was more prone to fracture at the subsurface and the elongation of the specimen decreased under external tensile loading [24]. As can be seen from Figs 11(c) and 14(c), the dimples in modified 316L specimen were more uniformly distributed than those in 316L specimen, but the dimples were of the same size.

**Table 4:** Tensile properties of 316L and modified 316L specimens.

Index	316L (1050°C)	Standard deviation of 316L	Modified 316L (1050°C)	Standard deviation of modified 316L
Tensile strength (MPa)	589	3.95	704	7.46
Yield strength (MPa)	260	6.92	454	7.66
Elongation at fracture (%)	72.83	4.43	57.45	6.45
Reduction of area (%)	77.72	10.23	65.98	11.94

### 3.5 Fatigue properties

**Fig 13:**
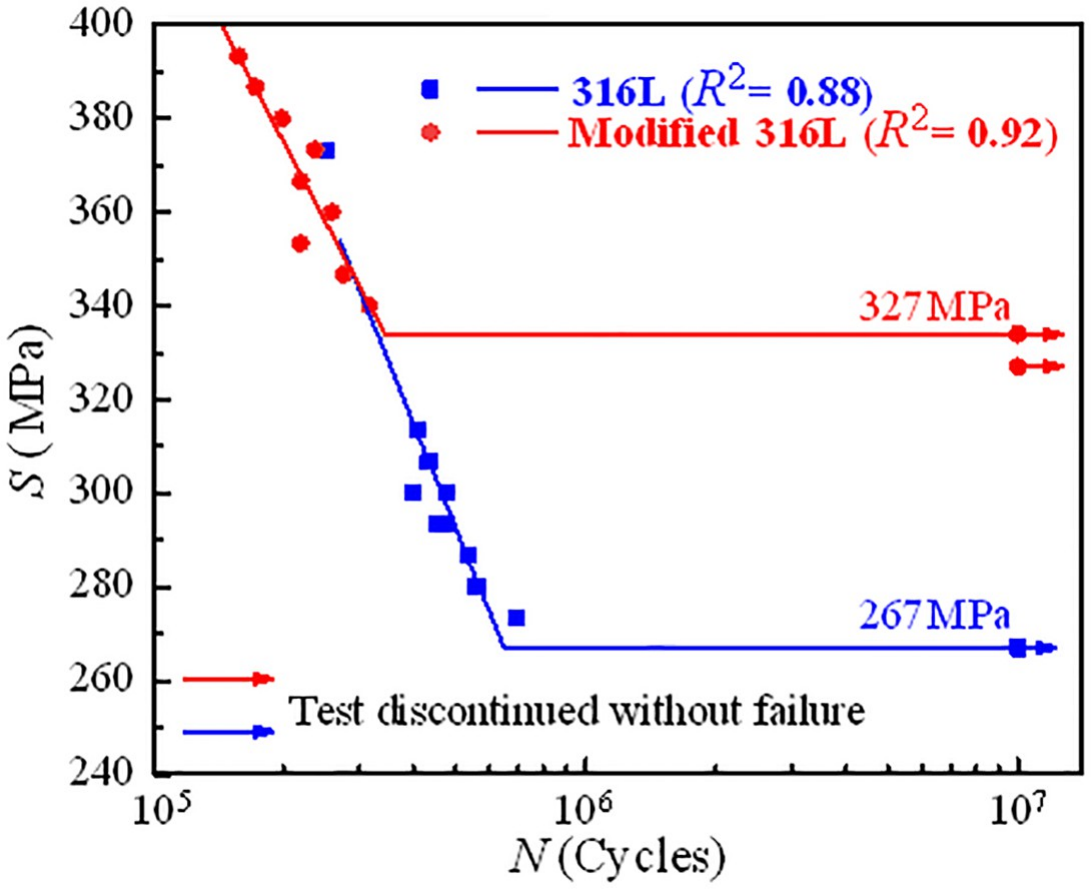
S-N curves of 316L and modified 316L specimens.

**Fig 14:**
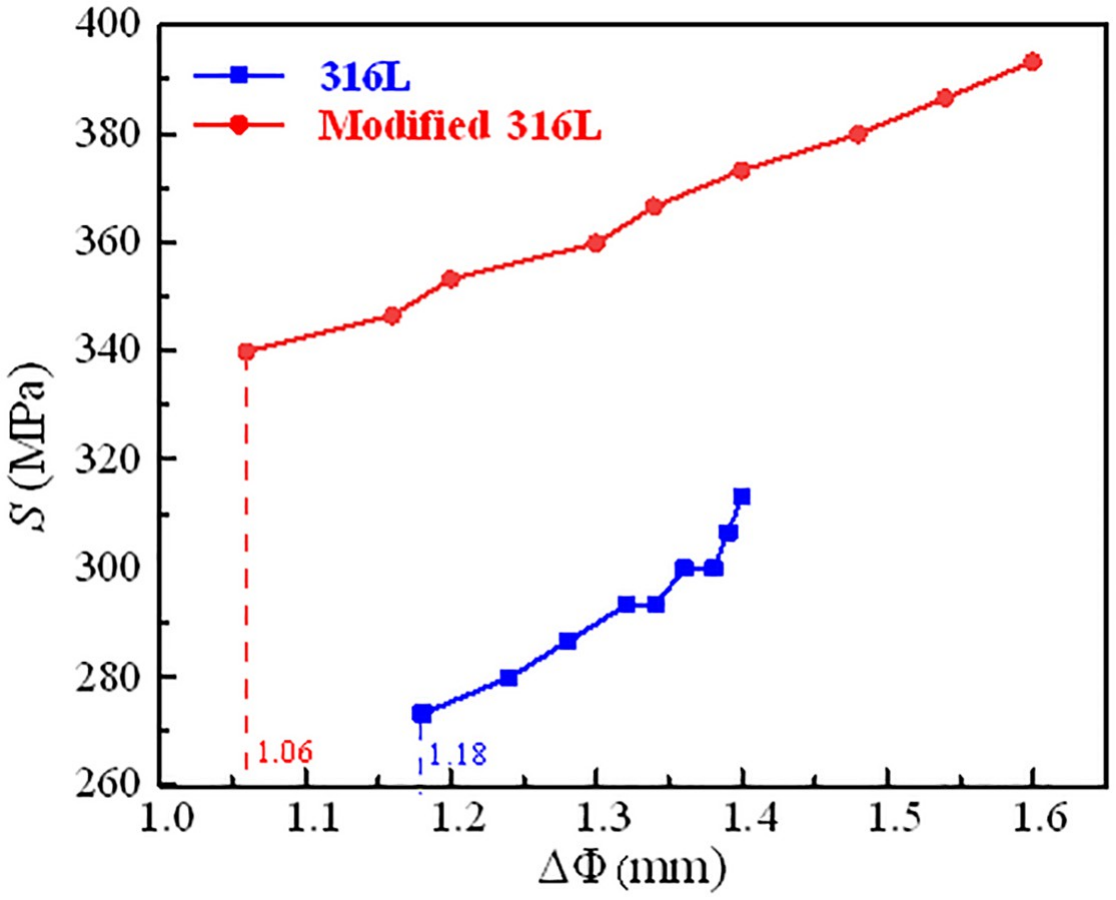
S-ΔΦ curves of 316L and modified 316L specimens.

The number of cycles (*N*) recorded after cyclic failure of the test specimen was the fatigue life of the specimen under the corresponding condition, or the number of cycles reached 10 million cycles and then the test was stopped, and the current state was recorded as a permanent fatigue state. The *S*-*N* curves of 316L and modified 316L specimens are shown in Fig 13. The fatigue strengths of 316L and modified 316L specimens were 267 MPa and 327 MPa, respectively. After UIT, the fatigue strength of the 316L increased by 22.47%, indicating that austenite grain refinement had a significant effect on the fatigue strength of 316L. However, when *S* was greater than 350 MPa, the fatigue life of modified 316L was not well strengthened, and even had a decreasing trend. The reason for this can be attributed to two aspects: at high stress range, the material was prone to plastic deformation, and the residual compressive stress caused by ultrasonic impact was released rapidly, leading to rapid crack sprouting in the modified 316L specimens; at low stress range, the residual compressive stress and high finish of the modified 316L surface can greatly inhibit crack initiation and extension, thus significantly improving the fatigue life [24].The fatigue strength to tensile strength ratios for 316L and modified 316L specimens were 0.45 and 0.46, respectively. Andrade et al. [27] and Hitoshi et al. [31] found that UIT can increase the fatigue strength of metallic materials by between 20% and 30%, and in a few cases, the fatigue limit can be increased by 50%, which was agreement with the results in this study. Fig 14 shows the relationship between the stress range and the difference in diameter after fracture (ΔΦ) of the specimens. ΔΦ increased with the increase of *S*. The diameter differences of 316L and modified 316L specimens at low stress range were 1.18 mm and 1.06 mm, respectively, which were both smaller than those of the permanent fatigue specimens. *R*^2^.

**Fig 15:**
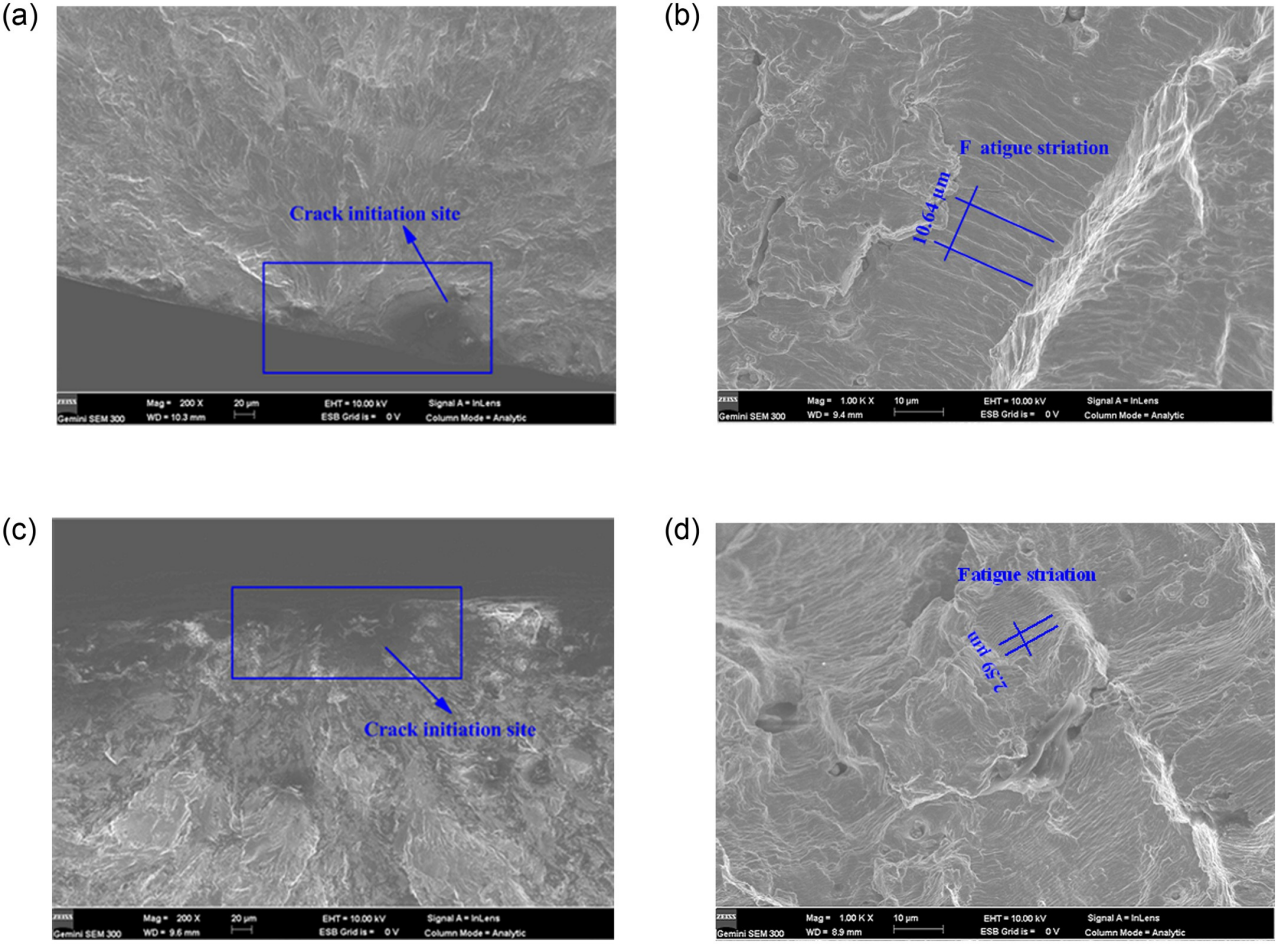
SEM fracture morphology of fracture surface of 316L specimens: (a) Crack source at *S* = 286.66 MPa; (b) Fatigue striation at *S* = 286.66 MPa; (c) Crack source at *S* = 313.34 MPa; (d) Fatigue striation at *S* = 313.34 MPa.

Fig 15 shows the fracture morphology of 316L specimens. From the crack sources in Fig 15(a) and 15(c), it can be seen that the crack initiation site was located on the surface of the specimen, and the crack sources were radial in shape at different stress ranges. The fatigue striation spacing is measured using Photoshop software, as shown in Fig 15(b) and 15(d). From Fig 15(b) and 15(d), it can be observed that the fatigue striation spacing is 3.54 μm at *S* = 286.66 MPa and it is 1.30 μm *S* = 313.34 MPa. When *S* increases by 9.31%, the fatigue striation spacing decreases by 63.28%, indicating that *S* has a greater effect on the fatigue striation spacing of 316L. The reason for this was that 316L specimens were more susceptible to the formation of slip bands of plastic deformation under cyclic loading, and the generation of slip bands provided favorable conditions for fatigue crack initiation [43]. The internal grains in austenitic steels were constrained by the surrounding neighboring grains and were therefore under triaxial stress. Grains near the surface were constrained only by grains deeper in the material and therefore experienced stress gradients and biaxial states of stress at the very surface. The number of slip systems activated in the "surface" grains was less than that activated in the "body" grains, so the former were more prone to plastic deformation. As a result, fatigue cracks in untreated specimens always started at the surface and then propagated internally until failure.

**Fig 16:**
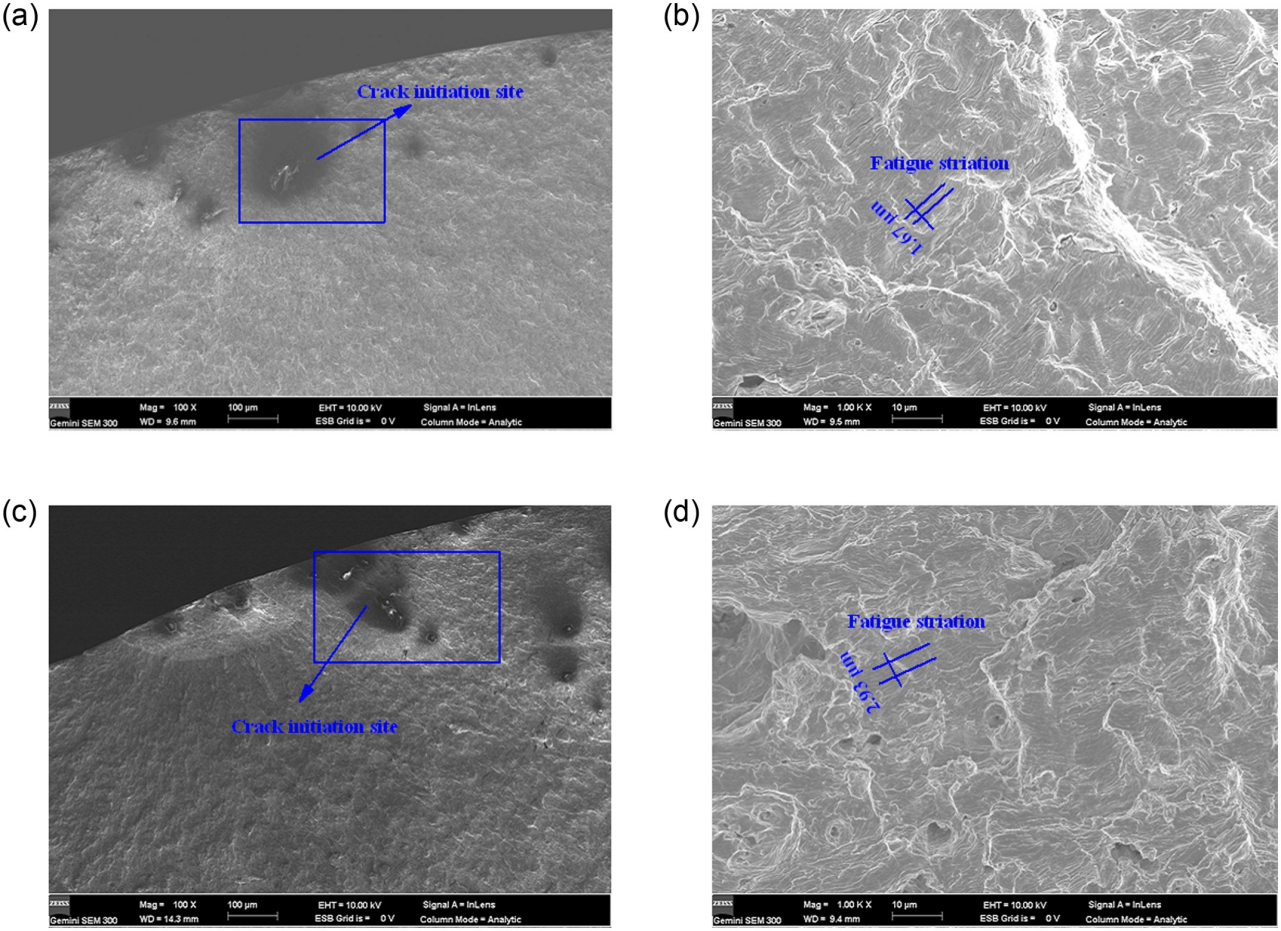
SEM fracture morphology of fracture surface of 316(UI) specimens: (a) Crack source at *S* = 353.34 MPa; (b) Fatigue striation at *S* = 353.34 MPa; (c) Crack source at *S* = 393.34 MPa; (d) Fatigue striation at *S* = 393.34 MPa.

Fig 16 shows the SEM fracture morphology of modified 316L specimens. At the low stress range, cracks sources of modified 316L specimens were far away from the surface of the specimen (Fig 16(a)), and they showed a radial pattern with small fatigue striation spacing. At the high stress range, cracks sources of modified 316L specimens were close to the surface of the specimens (Fig 16(c)). From Fig 16(b) and 16(d), it can be observed that the fatigue striation spacing was 0.84 μm at *S* = 353.34 MPa and it is 0.73 μm *S* = 393.34 MPa. When *S* increased by 11.13%, the fatigue striation spacing decreased by 13.10%, indicating that *S* had less effect on the fatigue striation spacing of modified 316L. Compared with the results of 316L, it can be found that the fatigue property of the modified 316L was better than that of 316L. The reason for this was that the specimen surface layer had fine grains, more dislocations, and high distortion energy, which prevented the expansion of brittle outer cracks, which led to delayed crack initiation, thus greatly increasing the fatigue life. Fatigue cracks nucleated at locations where internal stresses were concentrated, such as brittle inclusions. Under cyclic loading, debonding occurred at the interface between the inclusions and the matrix and the inclusions fracture, providing a site for fatigue crack initiation [44].

**Fig 17:**
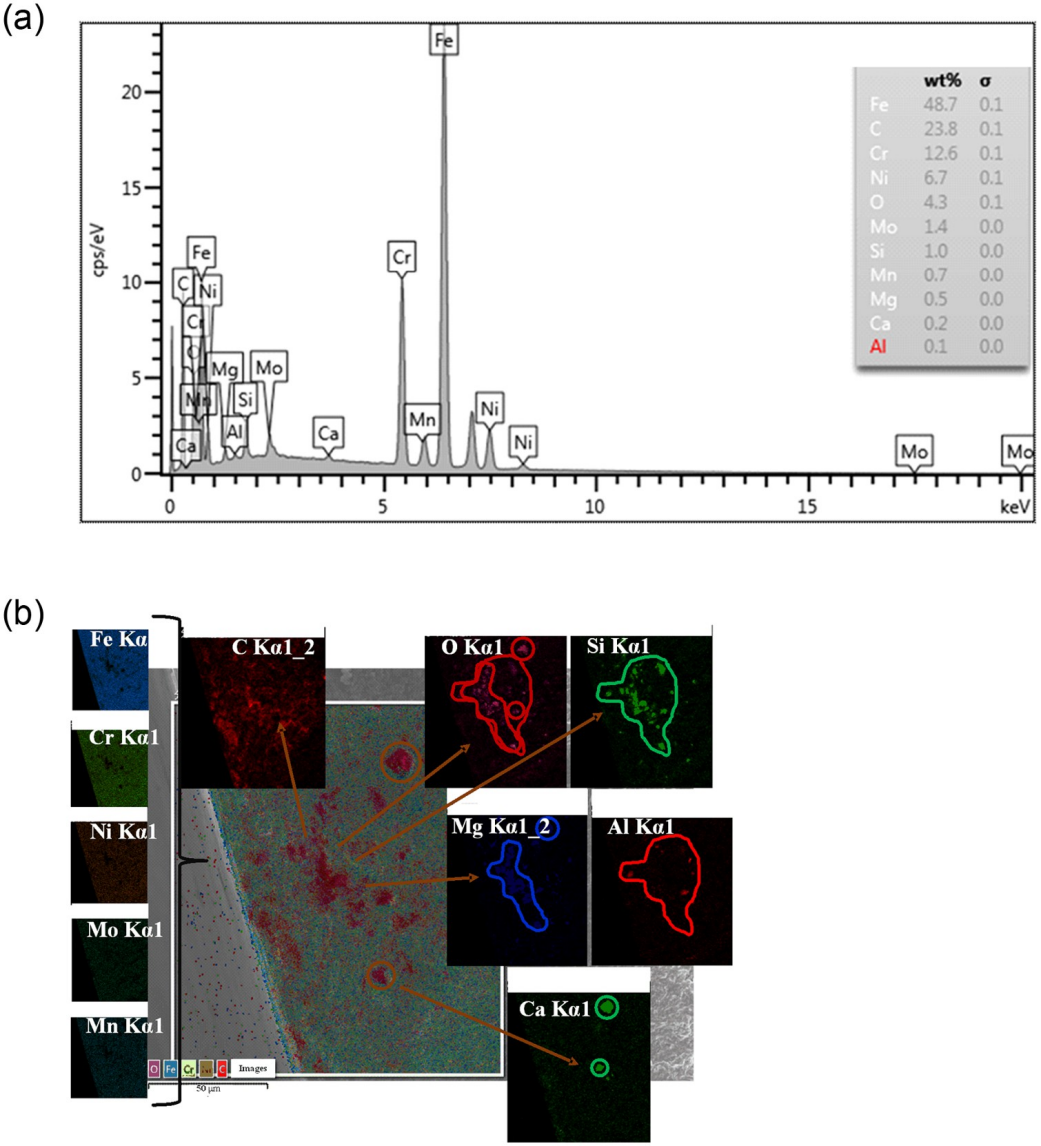
EDS analysis of crack source at S = 353.34MPa: (a) Chemical element content; (b) Chemical element distribution.

In conclusion, regardless of the stress level, the crack initiation site of modified 316L was located inside the specimen. The cracks started from the inside of modified 316L specimens. As the stress range decreased, the crack initiation site gradually moved toward the interior of the specimen. The crack source shown in Fig 16(a) was analyzed by x-ray spectrometer and the test results are shown in Fig 17. The material at the crack source was oxides dominated by SiO_2_. The cracks existed inside the inclusions and at the interface between the inclusions and the matrix.

**Fig 18:**
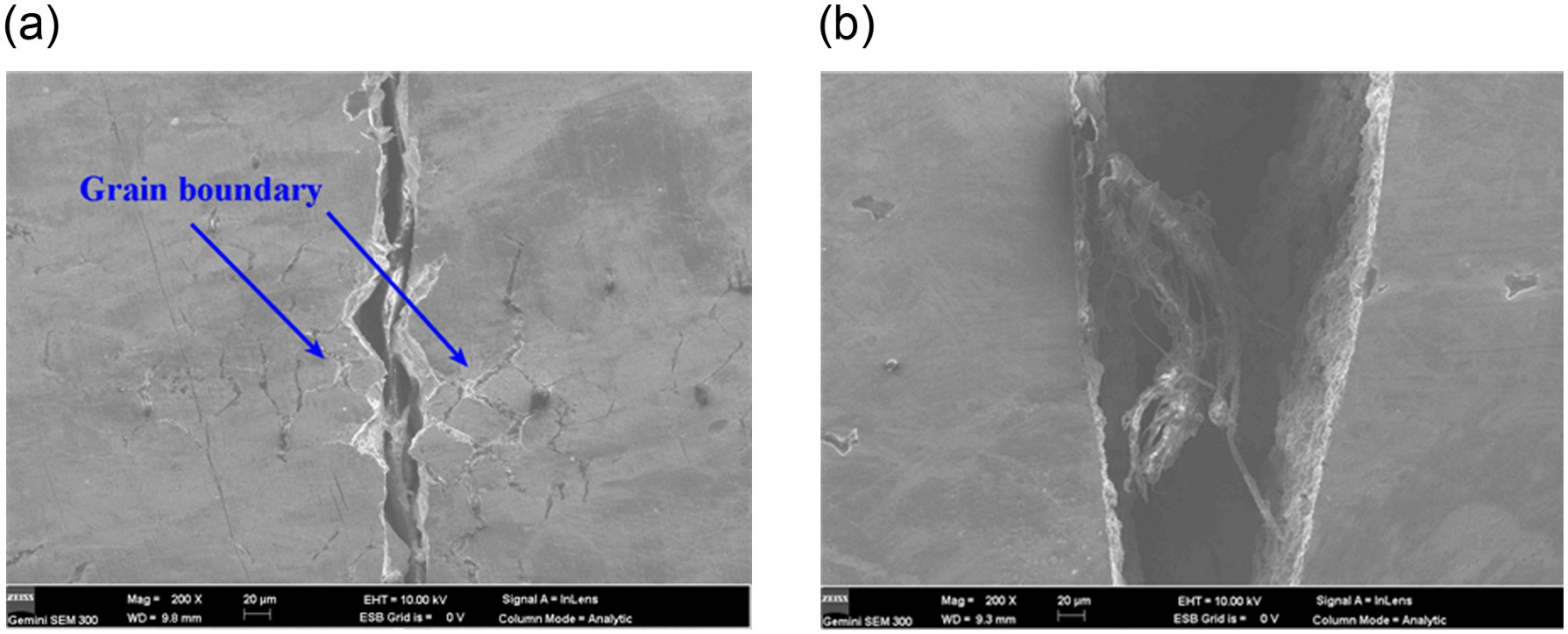
Fatigue cracks on the side of the fatigue fracture surface: (a) 316L at *S* = 286.66 MPa; (b) Modified 316L at *S* = 353.34 MPa.

As shown in Fig 18, the fatigue fracture surfaces of 316L and 316 (UI) specimens had a large number of crack sources on one side of the fracture surface. The crack sources in 316L specimens were at the austenitic grain boundaries. There were obvious austenitic features in the vicinity of the crack sources. The crack sources in modified 316L specimen were inside the specimen and were not evident on the surface of the specimen.

**Fig 19:**
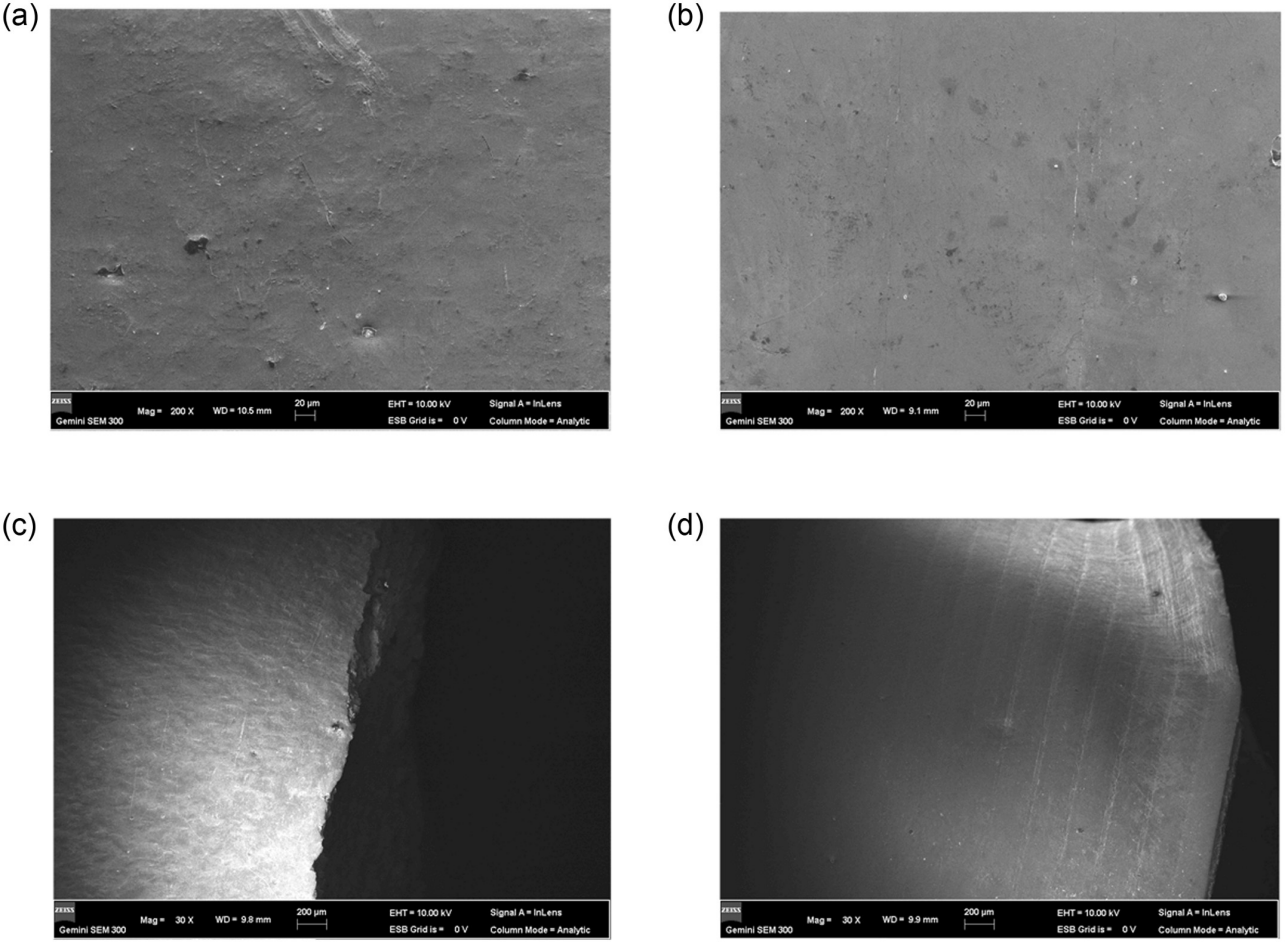
Fatigue fracture morphology of the side of the fatigue fracture surface: (a) Away from the fracture surface of 316L; (b) Away from the fracture surface of modified 316L; (c) Near the fatigue fracture surface of 316L; (d) Near the fatigue fracture surface of modified 316L.

The fatigue fracture morphology of the specimens on the side of the fatigue fracture surface is shown in Fig 19. 316L and modified 316L specimens showed no significant change on the side away from the fatigue fracture surface (Fig 19(a) and 19(b)). On the fatigue fracture surface, the 316L specimen had significant roughness on the surface and the fracture surface was jagged (Fig 19(c)), which was attributed to the slippage of the surface grains during sinusoidal loading of the specimens, resulting in very distinct austenite grain boundaries on the surface of 316L specimens. Modified 316L specimens had concentric rings on the fatigue fracture surface (Fig 19(d)), and the spacing of the concentric rings near the fracture surface was larger. This was due to the higher lattice energy and more dislocations on the surface of modified 316L specimens, which greatly improved the anti-slip ability of the grains, and the specimen surface exhibited concentric rings under cyclic loading conditions. It was worth noting that the above-mentioned features were absent in Fig 18.

### 3.6 Fatigue fracture mechanism

**Fig 20:**
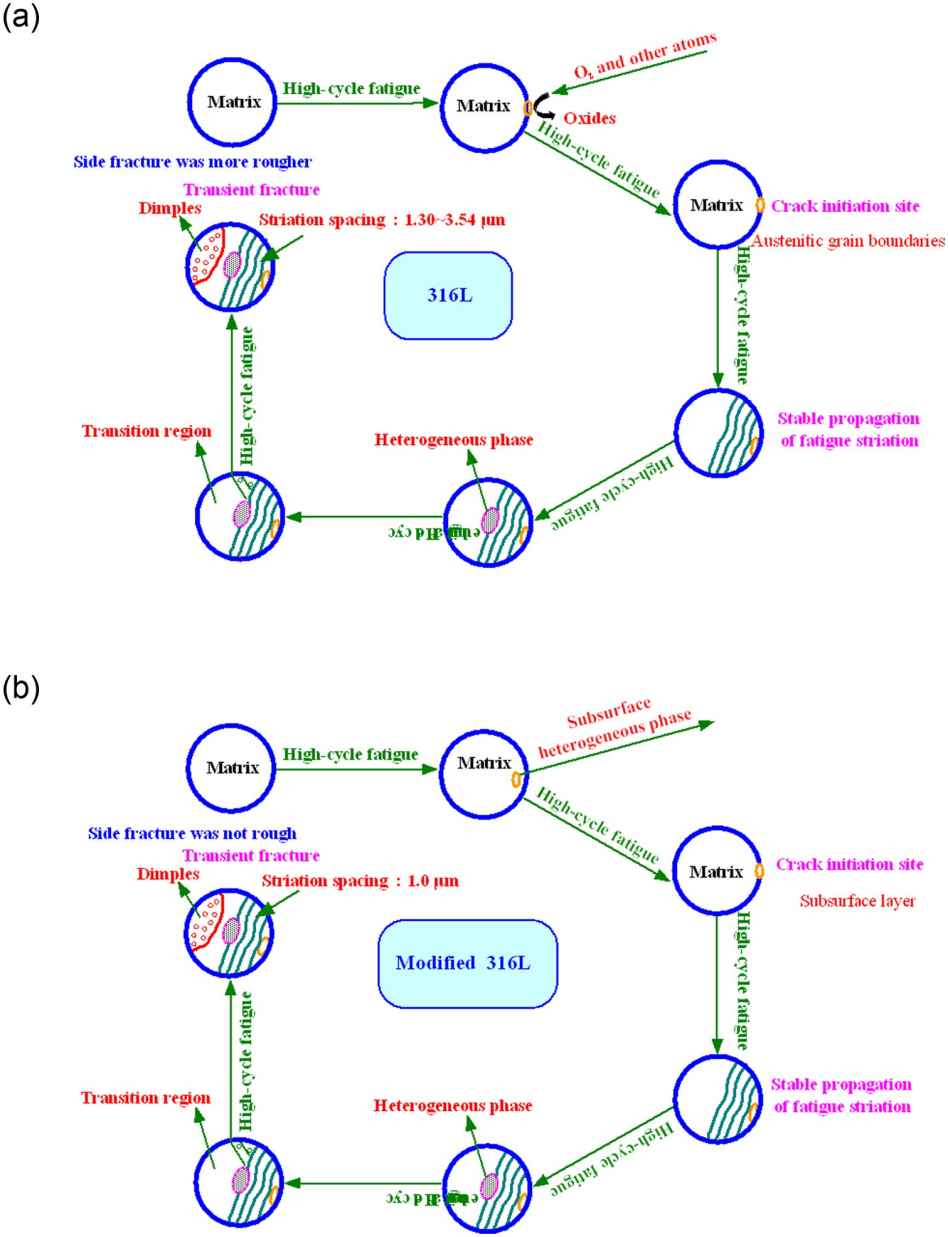
Fatigue fracture process model: (a) 316L; (b) Modified 316L.

The fatigue fracture mechanisms of 316L and modified 316L are shown in Fig 20. 316L and modified 316L differed in the location of the crack sources. The crack source in 316L came from the austenitic grain boundaries due to oxygen atoms and other atoms in the air crowding in and forming oxides on the surface of 316L. Modified 316L had a layer of fine crystals on the surface that effectively prevented oxygen atoms and other atoms in the air from crowding into the austenitic matrix. Its crack source was in the subsurface layer of modified 316L. As the number of fatigue cycles increased, fatigue cracks propagated steadily. When the crack propagated near the heterogeneous phase, the crack propagation rate fluctuated slightly. When the crack propagated into the transition zone, modified 316L specimens became extremely unstable, and crack propagation rate accelerated until the material instantaneously failed. The fatigue striation spacing of 316L was about 1.30~3.54, but that of modified 316L was less than 1 μm. Compared with modified 316L, the fracture was more zigzag, and the side surface after fracture was rougher.

Based on the above analysis, a method for detecting the degree of fatigue damage in 316L and modified 316L was proposed. For 316L, when the diameter of the 316L specimen in service was greater than the diameter of the specimen in the case of permanent fatigue, the specimen was safe for use. Conversely, the surface of the specimen can be observed using a portable OM. If the side surface of the specimen had significant roughness, the specimen should be included within the focus of testing. If micro cracks were observed on the side of the specimen, the specimen should be replaced immediately. For modified 316L, when the diameter of the modified 316L specimen in use was greater than the diameter of the specimen in the case of permanent fatigue, the specimen was safe to use. Conversely, the test should focus on the side surfaces of the specimen. When the concentric ring feature appeared on the side of modified 316L specimen, it should be included in the key inspection range and the maximum spacing between the concentric rings should be found, and then frequent observations should be made in the region of the maximum spacing. When macro cracks appeared in the region, the member should be replaced immediately.

## 4 Conclusions

Combining XRD test, tensile and fatigue tests, SEM and transmission electron microscopy investigations, the effect of UIT on the microstructural characterization and mechanical properties of 316L was investigated experimentally. The fatigue fracture mechanism of 316L before and after UIT was revealed. The main conclusions can be drawn as follows.

(1) The grain sizes of 316L and modified 316L were 27.5 μm and 9 μm, respectively. The surface grains of modified 316L showed a rheological trend. After UIT, 316L induced martensitic phase transformation.

(2) The tensile strengths of 316L and modified 316L specimens were 576 MPa and 703 MPa, respectively. After UIT, the tensile strength of the specimen increased by 22.04%, yield strength increased by 57.99%, and elongation at fracture and reduction of area were decreased by 18.72 and 13.04, respectively. The crack initiation site of 316L specimen was located at the surface, with inclusions of other materials or grain boundaries on the surface. The tensile fracture of modified 316L specimens originated from the subsurface layer. (3) Austenite grain refinement had a significant effect on the fatigue strength of 316L specimen. The fatigue strengths of 316L and modified 316L specimens were 267 MPa and 327 MPa, respectively. The fatigue strength to tensile strength ratios for 316L and modified 316L specimens were 0.47 and 0.46, respectively.

(4) The fatigue cracking of 316L started at the austenite grain boundaries, and the fatigue fracture surface was relatively rough. The fatigue crack sources in modified 316L originated from internal inclusions. The inclusions were oxides dominated by SiO_2_. As the stress range increased, the crack initiation site migrated to the interior and the fatigue fracture surface became flatter.

## Supporting information

S1 Dataset (ZIP)

## References

[1] Almangour B A. Additive manufacturing of high-performance 316L stainless steel nanocomposites via selective laser melting. University of California, Los Angeles, 2017.

[2] Lu K, Lu J. Surface Nanocrystallization (SNC) of Metallic Materials-Presentation of the Concept behind a New Approach. *Journal of Materials Science & Technology*, 1999, 15(3): 193–197.

[3] Amanov A, Cho I-S, Kim D-E, et al. Fretting wear and friction reduction of CP titanium and Ti–6Al–4V alloy by ultrasonic nanocrystalline surface modification. *Surface & Coatings Technology*, 2012, 207: 135–142.

[4] Cao X J, Pyoun Y S, Murakami R. Fatigue properties of a S45C steel subjected to ultrasonic nanocrystal surface modification. *Applied Surface Science*, 2010, 256(21): 6297–6303.

[5] Telang A, Gill A S, Tammana D, et al. Surface grain boundary engineering of Alloy 600 for improved resistance to stress corrosion cracking. *Materials Science & Engineering A*, 2015, 648: 280–288.

[6] Kahraman F. Surface layer properties of ultrasonic impact–treated AA7075 aluminum alloy. *Proceedings of the Institution of Mechanical Engineers*, *Part B*: *Journal of Engineering Manufacture*, 2018, 232(12): 2218–2225.

[7] Roland T, Retraint D, Lu K, et al. Enhanced mechanical behavior of a nanocrystallised stainless steel and its thermal stability. *Materials Science and Engineering*: *A*, 2007, 445: 281–288.

[8] Lei Y, Wang Z, Xu J, et al. Simultaneous enhancement of stress-and strain-controlled fatigue properties in 316L stainless steel with gradient nanostructure. *Acta Materialia*, 2019, 168: 133–142.

[9] Zhao X, Zhao D, Hu W, et al. Manufacturing of high-precision surface micro-structures on stainless steel by ultrasonic impact peening. *The International Journal of Advanced Manufacturing Technology*. 2021, 116(3–4): 1–16.

[10] Hansong C, Zhengye Z, Jianmin Z, et al. Effect of Ultrasonic Impact Strengthening on Surface Properties of 316L Stainless Steel Prepared by Laser Selective Melting. *Coatings*, 2022, 12(9): 1243.

[11] Wang X, Wang D. Microstructure and Mechanical Properties of Welded Joint of X80 Pipeline Steel before and after Ultrasonic Impact Treatment. *Journal of Materials Engineering and Performance*, 2021, 31(2): 1–13.

[12] Maria H, Karsten H, EVA H, et al. Effect of deep rolling on subsurface conditions of CoCr28Mo6 wrought alloy to improve the wear resistance of endoprostheses. *Journal of the Mechanical Behavior of Biomedical Materials*, 2021, 118: 104398. doi: 10.1016/j.jmbbm.2021.104398 33667927

[13] C B.S., W C.L., W L., et al. Effect of residual stress and phase constituents on corrosion-cavitation erosion behavior of 304 stainless steel by iso-material manufacturing of laser surface melting. *Journal of Materials Research and Technology*, 2023, 26: 6532–6551.

[14] Li L, Kim M, Lee S, et al. Influence of multiple ultrasonic impact treatments on surface roughness and wear performance of SUS301 steel. *Surface & Coatings Technology*, 2016, 307: 517–524.

[15] Wang G Q, Lei M K, Guo D M. Interactions between Surface Integrity Parameters on AISI 304 Austenitic Stainless Steel Components by Ultrasonic Impact Treatment. *Procedia CIRP*, 2016, 45: 323–326.

[16] Wang Z D, Sun G F, Lu Y, et al. Microstructural characterization and mechanical behavior of ultrasonic impact peened and laser shock peened AISI 316L stainless steel. *Surface & Coatings Technology*, 2020, 385: 125403.

[17] Yi Y, Yin F, Zhai J, et al. Microstructure Evolution and Numerical Modeling of TC4 Titanium Alloy during Ultrasonic Shot Peening Process. *Metals*, 2024, 14(3): 275

[18] Seob K M, Hu P S, Sik P Y, et al. Optimization of ultrasonic nanocrystal surface modification for surface quality improvement of directed energy deposited stainless steel 316L %J Journal of Materials Research and Technology [J]. 2020, 9(6): 15102–15122.

[19] Amanov A. Effect of local treatment temperature of ultrasonic nanocrystalline surface modification on tribological behavior and corrosion resistance of stainless steel 316L produced by selective laser melting. Surface & *Coatings Technology*. 2020, 398: 126080.

[20] Huang H, Wang Z, Yong X, et al. Enhancing torsion fatigue behaviour of a martensitic stainless steel by generating gradient nanograined layer via surface mechanical grinding treatment. *Materials Science and Technology*, 2013, 29(10): 1200–1205.

[21] Zhang K, Wang Z, Lu K. Enhanced fatigue property by suppressing surface cracking in a gradient nanostructured bearing steel. Materials Research Letters, 2017, 5(4): 258–266.

[22] Roland T, Retraint D, Lu K, et al. Fatigue life improvement through surface nanostructuring of stainless steel by means of surface mechanical attrition treatment. *Scripta Materialia*, 2006, 54(11): 1949–1954.

[23] Huang H, Wang Z, Lu J, et al. Fatigue behaviors of AISI 316L stainless steel with a gradient nanostructured surface layer. *Acta Materialia*, 2015, 87: 150–160.

[24] Jiaqiang D, Qinglong A, Guohui L, et al. Surface modification and its effect on the tensile and fatigue properties of 300M steel subjected to ultrasonic surface rolling process. *Surface and Coatings Technology*, 2021, 422: 127566.

[25] Liu Y, Wang D, Deng C, et al. Influence of re-ultrasonic impact treatment on fatigue behaviors of S690QL welded joints. I*nternational Journal of Fatigue*, 2014, 66: 155–160.

[26] Mordyuk B N, Prokopenko G I, Volosevich P Y, et al. Improved fatigue behavior of low-carbon steel 20GL by applying ultrasonic impact treatment combined with the electric discharge surface alloying. *Materials Science & Engineering A*, 2016, 659: 119–129.

[27] A M.F., P M.V., T M.C., et al. Fatigue life assessment in the very high cycle regime of AISI 316L stainless steel processed by L-DED additive manufacturing. *Procedia Structural Integrity*, 2022, 42: 1008–1016.

[28] Yan L, Gang L, Ni A, et al. Effect of ultrasonic rolled material layer on the corrosion fatigue resistance of railway axle EA4T alloy steel. *Engineering Failure Analysis*, 2024, 157: 107895.

[29] Zhou J, Retraint D, Sun Z, Kanouté P, et al. Comparative study of the effects of surface mechanical attrition treatment and conventional shot peening on low cycle fatigue of a 316L stainless steel. *Surface & Coatings Technology*, 2018, 349: 556–566.

[30] Pramod K, M G.S., S V., et al. Low cycle fatigue behaviour of Ti-13Nb-13Zr alloy in ultrasonic shot peened and stress relieved condition. *International Journal of Fatigue*, 2023, 166: 107289.

[31] Hitoshi S. Comparison between the improvements made to the fatigue strength of stainless steel by cavitation peening, water jet peening, shot peening and laser peening. *Journal of Materials Processing Tech*, 2018, 269: 65–78.

[32] Hong-Xiang Z, Yun L, Jing-Yu Z, et al. Comparison of strengthening effect and fatigue properties of SAF2205 cruciform welded joints by ultrasonic impact and cavitation jet peening treatment. *Materials Science & Engineering A*, 2022, 852: 143717.

[33] Chen X H, Lu J, Lu L, et al. Tensile properties of a nanocrystalline 316L austenitic stainless steel. *Scripta Materialia*, 2005, 52(10): 1039–1044.

[34] Kudryavtsev Y. Fatigue Improvement of Welded Elements by Ultrasonic Impact Treatment. *Experimental and Applied Mechanics*, 2011, 6: 75–87.

[35] Meng C, Jiang Y H, Yang J T, et al. The effect of combined laser bionic and ultrasonic impact on the microstructure and fatigue properties of AZ31B magnesium alloy TIG welded joints. *International Journal of Fatigue*, 2023, 176:107881.

[36] Wang C S, Li R F, Bi X L, et al. Microstructure and wear resistance property of laser cladded CrCoNi coatings assisted by ultrasonic impact treatment. *Journal of Materials Research and Technology*, 2023, 22: 853–864.

[37] NSPRC (National standards of People’s Republic of China). 2009. Metallic mechanicals-Tensile tests-Part 1: Test method. GB / T4340.1–2009. Beijing: China Standard Press.

[38] NSPRC (National standards of People’s Republic of China). 2010. Metallic mechanicals-Tensile tests-Part 1: Test methods at room temperature. GB / T228.1–2010. Beijing: China Standard Press.

[39] Wang D P, Song N X, Wang T, et al. Metal surface nanocrystallization by ultrasonic Processing. Journal of Tianjin University, 2007(2): 228–233.

[40] Erfan M, Nasim M, Alborz F, et al. Mechanical characterization and interfacial enzymatic activity of AISI 316L stainless steel after surface nanocrystallization. *Surface & Coatings Technology*, 2021, 405: 126729.

[41] Amir B, Morteza S, Aboozar T, et al. Enhanced surface properties and bioactivity of additively manufactured 316L stainless steel using different post-treatments. *Materials Today*: *Proceedings*, 2022, 70: 188–194.

[42] Mazánová V, Heczko M, Škorík V, et al. Microstructure and martensitic transformation in 316L austenitic steel during multiaxial low cycle fatigue at room temperature. *Materials Science and Engineering*: *A*, 2019, 767(8): 138407.

[43] Chan K S. Roles of microstructure in fatigue crack initiation[J]. International Journal of Fatigue, 2010, 32(9): 1428–1447.

[44] Peng Y, Liu Z, Chen C, et al. Effect of low-temperature surface hardening by carburization on the fatigue behavior of AISI 316L austenitic stainless steel[J]. Materials Science and Engineering: A, 2020, 769: 138524.

